# The supramolecular polymer-related signature predicts prognosis and indicates immune microenvironment infiltration in gastric cancer

**DOI:** 10.1016/j.clinsp.2025.100641

**Published:** 2025-04-13

**Authors:** Yan Liu, Hongyao Cui, Chuan Sun

**Affiliations:** Department of Gastroenterology, Ningbo Haishu People's Hospital, Ningbo, PR China

**Keywords:** Gastric cancer, Supramolecular polymer, LASSO, Nomogram, Immune landscape

## Abstract

**Background:**

Gastric Cancer (GC) remains a leading global cause of cancer mortality, underscoring the urgent need for advanced prognostic tools. This study aimed to construct and evaluate a prognostic risk signature based on Supramolecular Polymer-Related Genes (SPRGs) in gastric cancer.

**Methods:**

The authors downloaded data from TCGA-STAD, GEO, and CCLE databases for patients with GC and validation cohorts. Through consensus clustering, Cox proportional hazards models, LASSO Cox regression, and nomogram development, the authors identified and constructed a GC Prognostic risk Index (SPI). Additionally, the authors conducted drug sensitivity analysis and immune landscape assessment. Functional evaluations were conducted through colony formation, transwell invasion, and wound healing assays.

**Results:**

The authors identified that 182 SPRGs were significantly upregulated and 226 were downregulated in gastric cancer. Consensus clustering revealed two molecular subtypes, with cluster 1 having significantly lower overall survival compared to cluster 2. SPI effectively distinguished high-risk and low-risk patients across all cohorts. Furthermore, SPI was associated with tumor stage, lymph node metastasis, and tumor size, and could predict drug sensitivity in GC patients. Immune landscape analysis showed higher infiltration of naïve B cells, M2 macrophages, and activated NK cells in high-SPI patients. A nomogram model for GC prognosis using SPI and patient age was developed. KLC1 knockdown significantly suppressed GC cell proliferation, while markedly attenuating metastatic potential and invasion capacity.

**Conclusion:**

This study constructed a prognostic risk signature based on SPRGs in gastric cancer, which is closely related to clinical pathological features, drug sensitivity, and immune landscape, providing new insights for personalized treatment.

## Highlights

• Supramolecular Polymer-Related Genes (SPRGs) was associated with Gastric Cancer (GC).
• A prognostic signature was constructed based on SPRGs for GC patients.
• A nomogram was constructed for prognostic evaluation in GC patients.
• KLC1 was confirmed to promote proliferation, metastasis, and invasion in GC cells.

## Introduction

Gastric Cancer (GC) is a significant contributor to premature mortality, posing substantial healthcare and economic challenges. According to recent estimates, there will be approximately 26,890 new cases and 10,880 deaths from GC in the United States in 2024.^1^ While surgical intervention, chemotherapy, radiation, targeted therapies, and immunotherapy form the backbone of GC treatment, advanced disease stages at diagnosis, intrinsic heterogeneity, and resistance to chemotherapy contribute to a grim prognosis. The 25 % overall survival rate among GC patients underscores the urgent need for improved therapeutic approaches.^2^ The molecular and phenotypic diversity of the disease, coupled with a complex Tumor Microenvironment (TME), complicates treatment strategies. Classification systems, such as the Borrmann and Lauren histological classifications, and the TCGA's genetic-based categorization, offer insights into patient prognosis.^3^, ^4^, ^5^ However, the limited predictive power of the TCGA classification for drug response has hindered its clinical application. The advent of omics technologies has paved the way for multi-gene signatures, which hold promise for forecasting patient outcomes and guiding treatment strategies in GC. These advancements are crucial for enhancing the precision of therapeutic interventions and improving survival rates.

Supramolecular polymers are assemblies formed through non-covalent interactions such as hydrogen bonding, electrostatic forces, and van der Waals interactions.^6^ They exhibit unique properties such as self-healing, adaptability, and responsiveness to stimuli like pH, temperature, and light. These properties make them ideal for applications ranging from drug delivery systems to tissue engineering scaffolds.^7^ The dynamic nature of supramolecular polymers allows for reversible and tunable transitions, providing a robust foundation for developing sophisticated materials that can interact with biological systems in precise and controllable manners.^7^ While the application of supramolecular polymers in drug delivery and tissue engineering has been widely researched, their potential in genomic studies, particularly in developing prognostic signatures for cancers like GC, remains largely untapped.^8^ Research has demonstrated that Supramolecular Polymer-Related Genes (SPRGs) are deeply involved in the initiation and progression of cancer. Among these, Glial Fibrillary Acidic Protein (GFAP) plays a critical role in forming complex fibrous networks and is implicated in cellular signaling, intercellular communication, and cell migration processes, making it a potential biomarker for prostate cancer.^9^ In gastric cancer, Disrupted in Schizophrenia-1 (DISC1) exhibits regulatory functions in tumor immunity and is closely correlated with tumor histological type, staging, grading, and prognosis.^10^ However, to date, there has been no systematic investigation of SPRGs in gastric cancer.

This study aims to pioneer the use of SPRGs in constructing a prognostic signature for gastric cancer. Through comprehensive analysis and integration of genomic data, the authors seek to identify key genetic markers that, when modulated by supramolecular polymers, can accurately predict disease progression and response to therapy. This innovative approach not only addresses the current limitations in GC prognosis but also sets a precedent for future research at the interface of supramolecular chemistry and genomics.

## Materials and methods

### Data collection and processing

This present research is an observational study and follows the STROBE Statement. The authors downloaded bulk-seq datasets and corresponding clinical and prognostic data from the TCGA-STAD cohort. To ensure data quality and minimize potential bias, stringent exclusion criteria were applied. Patients were excluded if they met any of the following conditions: Incomplete clinical information (e.g., missing age, TNM stage, tumor size, or survival status); Follow-up time < 30 days, as extremely short follow-up periods may reflect immediate postoperative complications or non-cancer-related mortality rather than disease-specific outcomes; Ambiguous survival status (e.g., records marked as “lost to follow-up” or “unknown cause of death”). Ultimately, 341 GC patients were included as a training set for feature construction. Additionally, the authors downloaded GSE26253 and GSE84437 from the GEO database for model validation, and similar exclusion criteria were applied. GSE26899, GSE13861, GSE66229, and GSE29272 were also downloaded to validate the expression of feature-related genes. Expression data for feature-related genes in non-cancerous cell lines and esophagus/stomach cancer cell lines were obtained from the CCLE database. SPRGs in the GOCC_SUPRAMOLECULAR_POLYMER gene set were retrieved from the MSigDB database (Supplementary Materials: Table S1).

### Consensus clustering

The authors used the ConsensusClusterPlus package to perform consensus clustering on the TCGA-STAD cohort. Specifically, the authors extracted the transcriptome data of SPRGs from the cohort and performed Principal Component Analysis (PCA). The authors then processed the dataset using the ConsensusClusterPlus function, setting the maximum number of clusters to6, the clustering algorithm to partition around medoids, and the distance metric to Pearson correlation. The optimal number of clusters was determined using the Proportion of Ambiguous Clustering method. Kaplan-Meier (KM) survival curves were used to assess differences in overall survival between different subtypes.

### Risk signature construction and evaluation

Cox proportional hazards models were used to evaluate the association between SPRGs and overall survival in gastric cancer, selecting genes with *p* < 0.05 for further Least Absolute Shrinkage and Selection Operator (LASSO) Cox analysis. Genes with non-zero coefficients were selected to construct the SPI, calculated as follows: SPI=Σ(β_*i* × mRNA_i), where β_i is the coefficient of gene i, and mRNA_i is the expression level of gene i. All cohorts were classified into high-SPI and low-SPI groups according to the median risk score, and differences between groups were assessed using the KM survival curves. Receiver Operating Characteristic (ROC) curves were used to evaluate the accuracy of SPI in predicting overall survival.

### Drug sensitivity analysis

The authors used the pRRophetic package to analyze the sensitivity of TCGA-STAD patients to chemotherapeutic drugs, comparing the differences in drug sensitivity between high-SPI and low-SPI groups. Pearson correlation coefficients were calculated to assess the relationship between SPI and drug sensitivity.

### Immune landscape assessment

Multiple algorithms were used to evaluate the immune landscape of the TCGA-STAD cohort. CIBERSORT was used to calculate immune cell infiltration, and ESTIMATE was used to calculate stromal score, immune score, and tumor purity. Pearson correlation coefficients were calculated to assess the relationship between SPI and immune cell infiltration, and differences in immune cell infiltration between high-SPI and low-SPI patients were compared.

### Nomogram development

Multivariate Cox proportional hazards models were used to identify independent prognostic factors for overall survival in the TCGA-STAD cohort. The rms package was utilized to build a nomogram model. Calibration curves, decision curves, and ROC curves were used to evaluate the performance of the nomogram in predicting overall survival in GC patients.

### Differential expression and enrichment analysis

The limma package was used for differential expression analysis, selecting genes with adjusted p-value < 0.05 and |log2(fold change)| > 1 for Gene Ontology (GO) annotation and KEGG pathway enrichment analysis. Enrichment analysis was performed using the clusterProfiler package. Additionally, hallmark gene sets from the MSigDB database were analyzed for gene set enrichment using the results of differential expression analysis.

### Somatic mutation analysis

Somatic mutations in the TCGA-STAD cohort were analyzed using the maftools package, and the Tumor Mutation Burden (TMB) and its correlation with SPI were assessed. Differences in overall survival between high-TMB and low-TMB groups were assessed using KM curves.

### Cell culture

The GC cell lines MKN45 and the normal gastric epithelial cell line GES-1 were selected for this study. All cell lines were authenticated and tested regularly for mycoplasma contamination. MKN45 and HGC-27 were cultured in RPMI 1640 medium (Gibco), while GES-1 was maintained in DMEM (Gibco). Both media were supplemented with 10 % fetal bovine serum (FBS, Gibco), 100 U/mL penicillin, and 100 µg/mL streptomycin. Cells were incubated at 37 °C in a humidified atmosphere containing 5 % CO₂. For experiments, cells in the logarithmic growth phase were used, ensuring they were grown as adherent monolayers.

### KLC1 gene silencing

Three specific siRNAs targeting KLC1 mRNA were designed and synthesized by Guangzhou RiboBio Co., Ltd., with scramble siRNA serving as the negative control. Transfection was performed using Lipofectamine 3000 (Invitrogen) according to the manufacturer's instructions. Logarithmically growing cells were transfected with the siRNA-lipid complex, and cells were collected 48 h post-transfection for subsequent experiments. The efficiency of KLC1 silencing was validated by RT-qPCR and Western blot analysis to measure mRNA and protein expression levels, respectively.

### Western blot analysis

For protein expression analysis, total proteins were extracted from logarithmically growing cells using RIPA lysis buffer (Sigma-Aldrich) supplemented with a protease inhibitor cocktail. Protein concentrations were determined using the BCA Protein Assay Kit (Thermo Fisher Scientific). Equal amounts of protein (30 µg) were separated by 10 % SDS-PAGE and transferred onto PVDF membranes (Millipore). Membranes were blocked with 5 % non-fat milk in TBST for 1 h at room temperature and then incubated overnight at 4 °C with primary antibodies against KLC1 (Santa Cruz Biotechnology, 1:1000 dilution) and GAPDH (Cell Signaling Technology, 1:5000 dilution). After washing, membranes were incubated with HRP-conjugated secondary antibodies (1:5000 dilution) for 1 h at room temperature. Proteins were visualized using ECL chemiluminescence reagents (Pierce), and images were captured using a chemiluminescent imaging system. Band intensities were quantified using ImageJ software, and relative protein expression levels were normalized to GAPDH.

### Gene expression detection

Total RNA was extracted from cells using TRIzol reagent (Invitrogen), and its concentration and purity were assessed using a NanoDrop spectrophotometer (A260/A280 ratio between 1.8 and 2.0). One microgram of total RNA was reverse-transcribed into cDNA using the PrimeScript RT Reagent Kit (Takara). Quantitative real-time PCR (RT-qPCR) was performed using SYBR Green Premix (Roche) with the following primers: KLC1 forward 5′-TGGCTATCCGTGAGAAAACC-3′ and reverse 5′-CTCCACCTCCTCGTACTTGC-3′; GAPDH forward 5′-CGGAGTCAACGGATTTGGTCGTAT-3′ and reverse 5′-AGCCTTCTCCATGGTGGTGAAGAC-3′ The thermal cycling conditions consisted of 40 cycles at 95 °C for 10 s and 60 °C for 30 s. Relative expression levels were calculated using the 2^^-ΔΔCt^ method.

### Colony formation assay

Cells from control and KLC1-silenced groups were seeded at a density of 500 cells per well in 6 well plates and cultured for 10 days. Colonies were fixed with 4 % paraformaldehyde, stained with 0.1 % crystal violet, and counted if they contained >50 cells.

### Transwell invasion assay

Transwell chambers (Corning, 8 μm pore size) were coated with Matrigel. A total of 1 × 10⁵ cells suspended in serum-free medium were added to the upper chamber, while the lower chamber contained medium supplemented with 20 % FBS as a chemoattractant. After 24 h, invaded cells were fixed with 4 % paraformaldehyde, stained with 0.1 % crystal violet, and counted under a microscope in five random fields.

### Wound healing assay

Cells were grown to confluence in 6-well plates, and a scratch was made using a 200 μL pipette tip. Detached cells were removed by PBS washing. Images were captured at 0 and 24 h, and wound healing rates were calculated using ImageJ software.

### Statistical analysis

Data analysis and visualization were performed using *R* version 4.4.1. Comparisons between groups were analyzed using unpaired *t*-tests, Wilcoxon test, or one-way ANOVA, as appropriate, and correlations were assessed using Pearson correlation analysis. Kaplan-Meier analysis was used to evaluate survival; p-values <0.05 were considered statistically significant.

## Results

### SPRGs and heterogeneity in gastric cancer

**Fig. 1:**
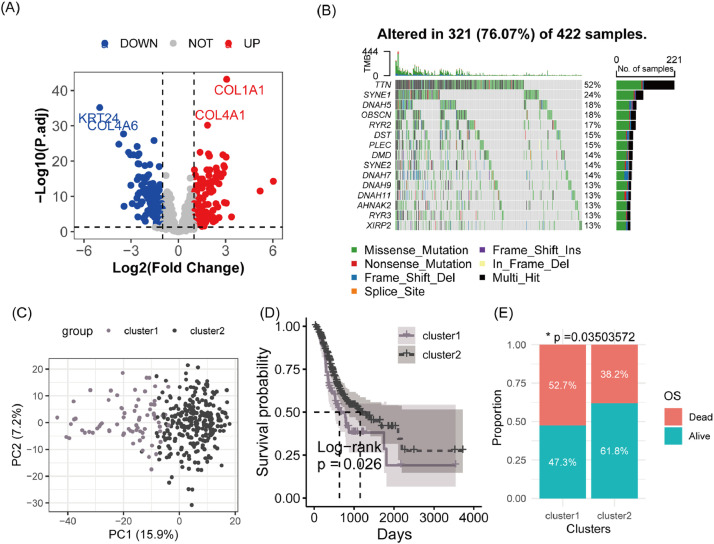
Analysis of molecular heterogeneity of gastric cancer associated with SPRGs. (A) Volcano plot of differential expression of SPRGs in the TCGA-STAD cohort. This plot highlights genes that are significantly up- or downregulated. (B) Somatic mutation characteristics of SPRGs in the TCGA-STAD cohort. A summary of the mutation frequencies and types observed in SPRGs. (C) Principal Component Analysis (PCA) plot comparing cluster 1 and cluster 2 subtypes. PCA is used to visualize the variance between the two clusters. (D) Kaplan-Meier survival curves for cluster 1 and cluster 2. These curves compare the overall survival between the identified molecular subtypes. (E) Comparison of survival outcomes between cluster 1 and cluster 2 subtypes. Statistical analysis demonstrating differences in patient outcomes between the two clusters.

To explore the relationship between SPRGs and the molecular heterogeneity of gastric cancer, the authors first analyzed their differential expression in the TCGA-STAD cohort. The authors found that 182 SPRGs were significantly upregulated and 226 were downregulated in GC (Fig. 1A). The authors also observed widespread somatic mutations in SPRGs in GC (Fig. 1B). These results suggest that SPRGs are closely associated with the molecular heterogeneity of gastric cancer. Therefore, the authors performed consensus clustering on the TCGA-STAD cohort based on SPRGs, identifying two molecular subtypes (cluster 1 and cluster 2) (Fig. S1). PCA showed clear boundaries between these two subtypes (Fig. 1C). Survival analysis revealed that cluster 1 had significantly lower overall survival compared to cluster 2 (*p* = 0.026, Fig. 1D). Additionally, cluster 1 has a notably higher proportion of deceased patients than cluster 2 (*p* = 0.035, Fig. 1E). These findings indicate that the heterogeneous expression of SPRGs in GC is associated with overall survival.

### A novel prognostic indicator for gastric cancer: SPI

**Fig. 2:**
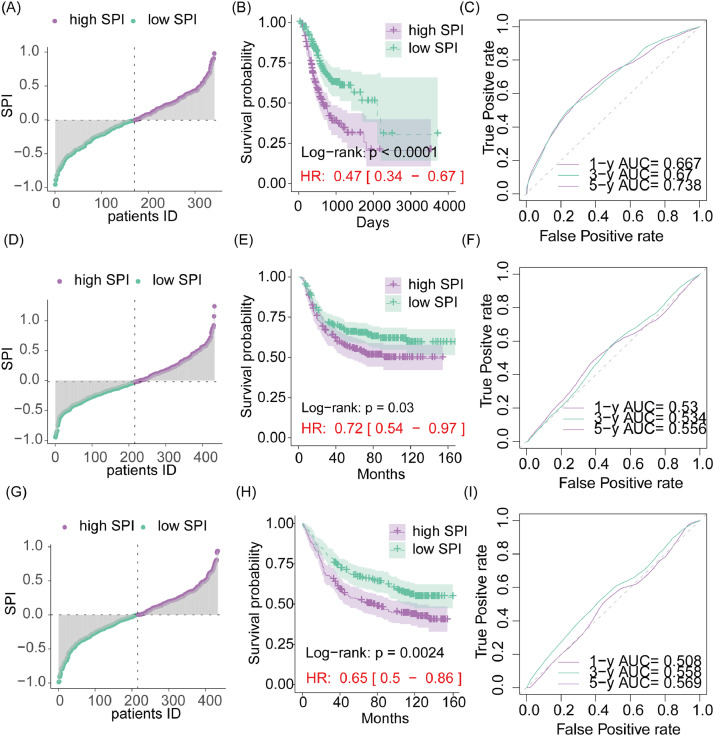
Risk signature constructed by lasso cox regression using SPRGs for gastric cancer patients. (A) Stratification of the TCGA-STAD cohort according to risk score. Patients are divided into high-risk and low-risk groups based on their risk scores. (B) Kaplan-Meier survival curve analysis between groups. Comparison of survival outcomes between the high-risk and low-risk groups. (C) Receiver Operating Characteristic (ROC) analysis of the risk score. Evaluation of the predictive accuracy of the risk score for overall survival. (D‒F) Similar analyses as (A‒C) but performed on the GSE26253 and GSE84437 cohorts.

Univariate Cox analysis identified 136 SPRGs significantly associated with overall survival in GC (Supplementary Materials Table S2). Further LASSO Cox analysis selected 11 genes with non-zero coefficients (Fig. S2, Table S3). The final SPI was constructed as follows: −0.01474 * KEAP1 + 0.14971 * SRI - 0.05824 * MFAP1 + 0.17692 * NRP1 + 0.07341 * KLC1 + 0.06557 * MFAP2 - 0.02490 * INCENP - 0.14048 * KIF21B + 0.07353 * MYOZ3 + 0.02361 * DST + 0.07176 * TIAM1. To evaluate the role of SPI in GC prognosis, the authors divided all cohorts into high-SPI and low-SPI groups based on the median risk score (Fig. 2A, D, and G) and performed KM survival curve analysis. The clinicopathological characteristics of the two groups are presented in Table S4. Results showed that high-SPI patients had significantly lower overall survival compared to low-SPI patients across all cohorts (Fig. 2B, E, and H). ROC analysis indicated that SPI had an accuracy of 0.667, 0.67, and 0.738 for predicting 1-, 3-, and 5-year overall survival in the TCGA-STAD cohort (Fig. 2C), 0.53, 0.534, and 0.556 in the GSE26253 dataset (Fig. 2F), and 0.508, 0.558, and 0.569 in the GSE84437 dataset (Fig. 2I).

### SPI-driven gene expression patterns in gastric cancer

**Fig. 3:**
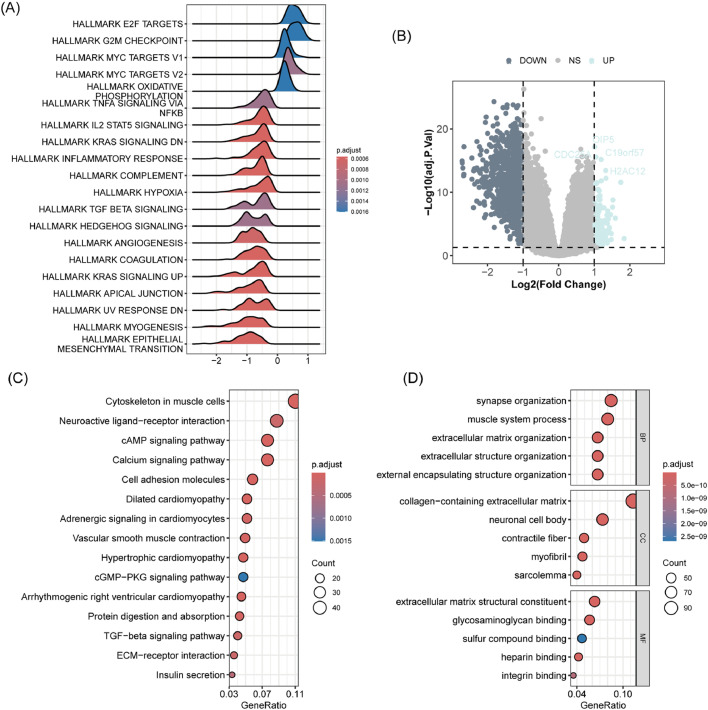
Gene expression patterns driven by the SPRGs-related risk signature. (A) Gene Set Enrichment Analysis (GSEA) plot. It displays the enrichment scores of various Hallmark gene sets in gastric cancer. (B) Volcano plot of differential gene expression. This plot identifies genes that are significantly differentially expressed between high-risk and low-risk groups. (C) Bubble chart of pathway enrichment analysis. It visualizes the pathways enriched among differentially expressed genes. (D) Bubble chart of GO enrichment analysis. It presents the Gene Ontology terms enriched among differentially expressed genes.

Gene Set Enrichment Analysis (GSEA) revealed significant differences in hallmark gene sets between high-SPI and low-SPI groups, with E2F targets, G2M checkpoint, and MYC target-related gene sets being activated in high-SPI patients, while hypoxia, inflammatory response, angiogenesis, and coagulation were significantly suppressed (Fig. 3A). Furthermore, the authors identified 1225 Differentially Expressed Genes (DEGs) (Fig. 3B), which were significantly enriched in multiple signaling pathways, including cAMP, calcium, cGMP-PKG, and TGF-beta signaling (Fig. 3C). GO annotation analysis showed that these DEGs were associated with synapse organization, muscle system processes, and extracellular matrix organization (Fig. 3D).

### Somatic mutation characteristics associated with SPI

**Fig. 4:**
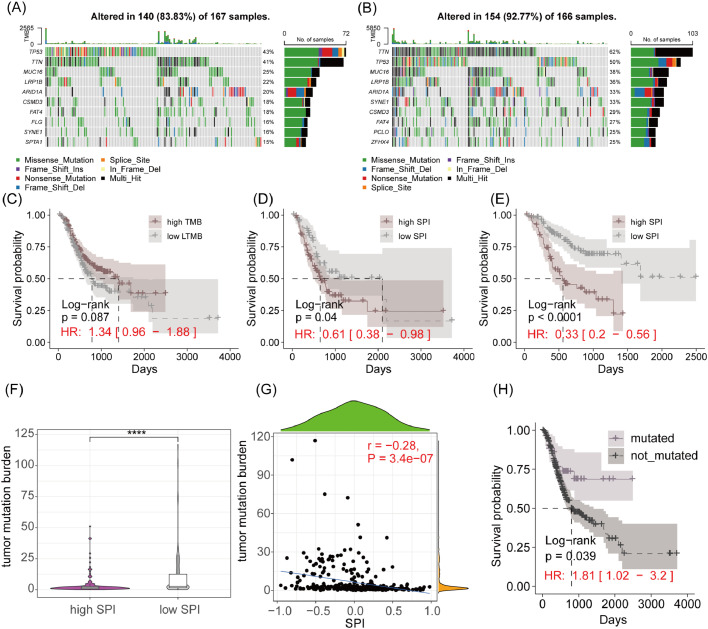
Somatic mutation landscape associated with risk scores. (A‒B) Top 10 Most Mutated Genes in High-Risk and Low-Risk Groups. Bar plots showing the frequency of mutations in the top 10 genes for each group. (C) Kaplan-Meier Survival curve analysis between high and low Tumor Mutational Burden (TMB) Groups. Comparison of survival outcomes based on TMB levels. (D‒E) Kaplan-Meier survival curve analysis within high and low TMB groups. Further stratification of survival outcomes within each TMB category by risk score. (F) Comparison of TMB differences between high-risk and low-risk groups. Box plot illustrating the distribution of TMB in the two risk groups. (G) Pearson correlation analysis between risk score and TMB. Scatter plot showing the relationship between the risk score and TMB. (H) Kaplan-Meier survival curve analysis for mutated vs. Non-mutated NRXN1 gene. Comparison of survival outcomes based on the mutation status of a specific gene. **** *p* < 0.0001.

Fig. 4A and B show the somatic mutation profiles of high-SPI and low-SPI GC patients in the TCGA-STAD cohort. The distribution of frequently mutated genes was similar between the two groups. Survival analysis revealed that patients in the high-TMB group had longer overall survival compared to those in the low-TMB group (Fig. 4C). Additionally, high-SPI patients in both high-TMB and low-TMB groups had significantly lower overall survival compared to low-SPI patients (Fig. 4D and E). Low-SPI patients had higher TMB compared to high-SPI patients (Fig. 4F), and TMB showed a significant negative correlation with SPI (Fig. 4G). Notably, NRXN1 mutations were associated with significantly better overall survival in GC patients (Fig. 4H).

### Association between SPI and clinical pathological features

**Fig. 5:**
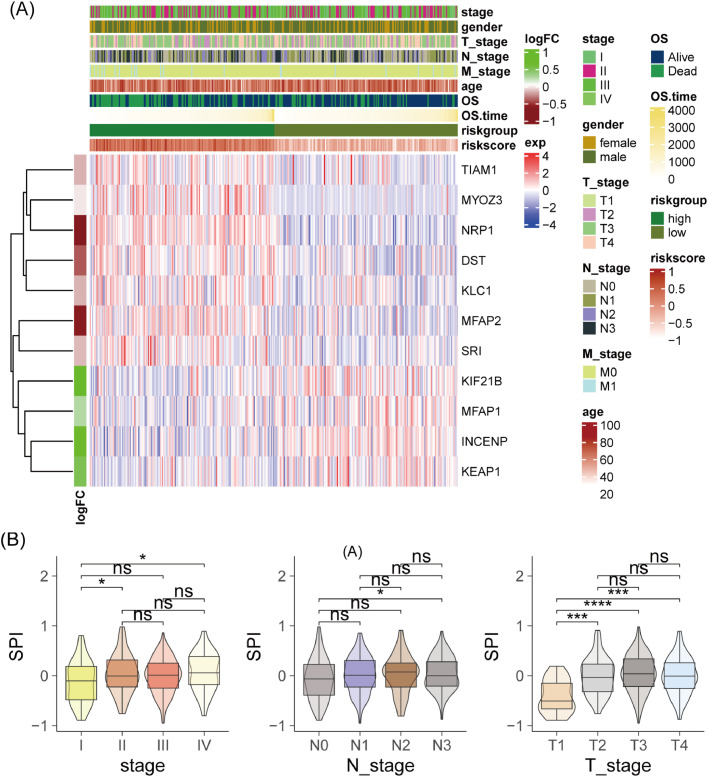
Correlation analysis between risk score and clinicopathological features. (A) Heatmap of SPRG expression related to risk score. Visualization of the expression patterns of SPRGs associated with the risk score. (B) Comparison of risk scores among different stages, N Stages, and T Stages. Box plots showing the distribution of risk scores across various clinical stages. ns, not significant; * *p* < 0.05; *** *p* < 0.001; **** *p* < 0.0001.

The authors visualized the expression heatmap of SPI-related genes in the TCGA-STAD cohort, showing that TIAM1, NRP1, MYOZ3, KLC1, DST, SRI, and MFAP2 were downregulated in the low-SPI group, while KIF21B, MFAP1, and KEAP1 were upregulated (Fig. 5A). Moreover, patients with stages II and IV had higher SPI compared to those with stage I (*p* < 0.05), and patients with N3 stage had higher SPI compared to those with N0 stage (*p* < 0.05). Similarly, patients with T2 (*p* < 0.001), T3 (*p* < 0.0001), and T4 (*p* < 0.001) stages had higher SPI compared to those with the T1 stage (Fig. 5B). These results indicate that SPI is associated with tumor stage, lymph node metastasis, and tumor size.

### SPI as an indicator of drug sensitivity in gastric cancer

**Fig. 6:**
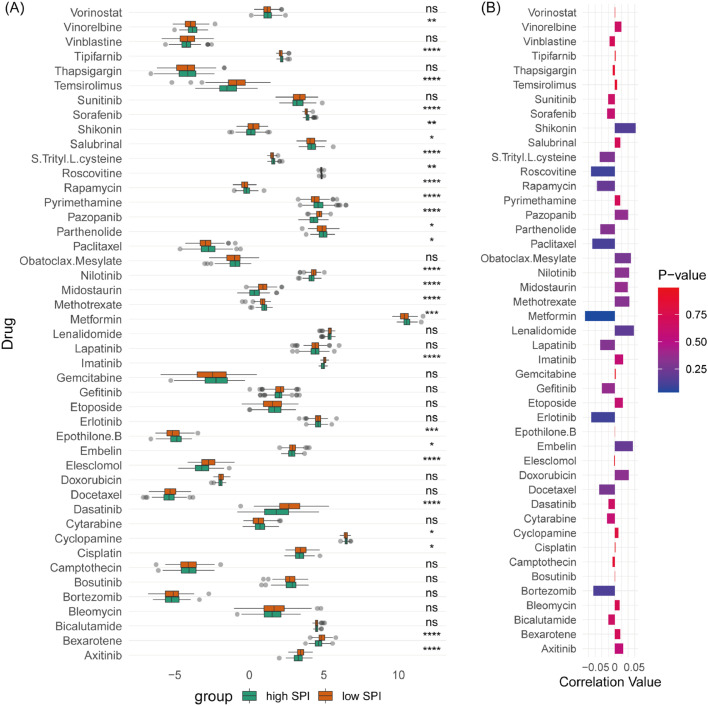
Indication of drug sensitivity by risk score in gastric cancer patients. (A) Comparison of chemotherapy drug sensitivity between high-risk and low-risk groups in the TCGA-STAD cohort. Bar plots representing the IC50 values or other measures of drug sensitivity. (B) Correlation between drug sensitivity and risk score. Scatter plot or correlation matrix illustrating the association between drug sensitivity and the risk score. ns, not significant; * *p* < 0.05; ** *p* < 0.01; *** *p* < 0.001; **** *p* < 0.0001.

The authors assessed the sensitivity of TCGA-STAD patients to 45 drugs, finding significant differences in sensitivity to 26 drugs between high-SPI and low-SPI patients (Fig. 6A). Low-SPI patients were more sensitive to metformin (Glucophage), erlotinib, roscovitine, and paclitaxel, whereas high-SPI patients presented higher sensitivity to shikonin, embelin, and lenalidomide. Pearson correlation analysis showed complex relationships between SPI, its related SPRGs, and drug sensitivity (Figs. 6B and S4).

### Tumor immune microenvironment in different SPI patients

**Fig. 7:**
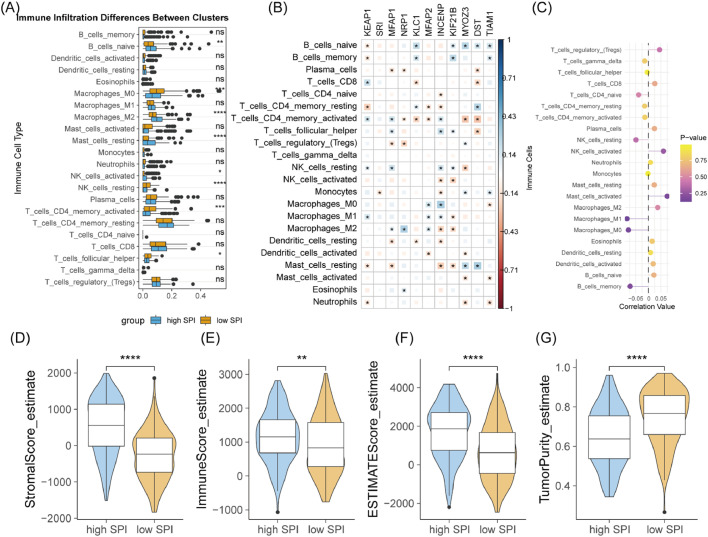
Association between risk score and tumor immune landscape. (A) Comparison of immune cell infiltration between high-risk and low-risk groups in the TCGA-STAD cohort. Violin or box plots showing the infiltration levels of immune cells. (B) Pearson correlation analysis between SPRG expression and immune cell infiltration. Scatter plots or heatmaps showing the correlation between SPRG expression and immune cell infiltration. (C) Pearson correlation analysis between risk score and immune cell infiltration. Similar to (B), but focusing on the risk score instead of individual SPRGs. (D‒G) Comparison of stromal score, immune score, ESTIMATE score, and tumor purity between high-risk and low-risk groups. Box plots displaying the distribution of these metrics in the two risk groups. ns, not significant; * *p* < 0.05; ** *p* < 0.01; *** *p* < 0.001; **** *p* < 0.0001.

The authors calculated the cell fraction of 22 immune cell types for the TCGA-STAD cohort. High-SPI patients had higher infiltration of naive B-cells, M2 macrophages, and activated NK cells, while low-SPI patients had higher infiltration of M0 macrophages, resting NK cells, activated CD4 memory T-cells, and follicular helper T-cells (Fig. 7A). Pearson correlation coefficients between SPI-related genes and immune cell infiltration are shown in Fig. 7B and C. Additionally, ESTIMATE scores revealed that low-SPI patients had lower stromal score, immune score, and ESTIMATE score, and higher tumor purity compared to high-SPI patients (Fig. 7D–G).

### Development of a nomogram model based on SPI for gastric cancer

**Fig. 8:**
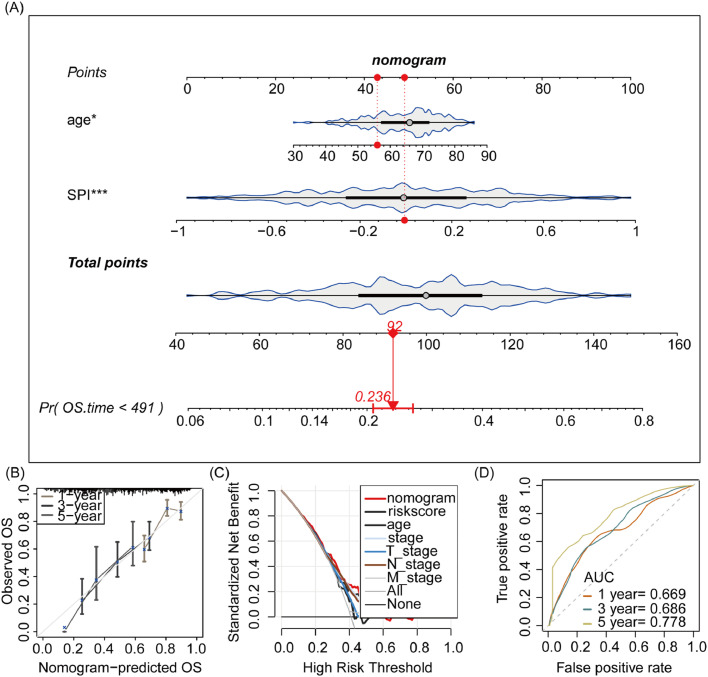
Construction of a nomogram integrating risk score and age for predicting overall survival in gastric cancer patients. (A) Nomogram. A graphical tool integrating SPI and age to estimate the probability of overall survival. (B) Calibration plot. Assessment of the nomogram's accuracy in predicting survival probabilities against observed survival rates. (C) Decision curve analysis. Evaluation of the clinical utility of the nomogram by comparing the net benefit of using the model for different threshold probabilities of high-risk classification. (D) Receiver Operating Characteristic (ROC) curve. Display of the diagnostic ability of the nomogram for 1-, 3-, and 5-year overall survival.

Multivariate Cox analysis identified SPI and age as independent prognostic factors for overall survival in GC (Table S5). Therefore, the authors constructed a nomogram model based on SPI and age (Fig. 8A) and evaluated its performance. Calibration analysis (Fig. 8B) demonstrated good alignment between the nomogram-predicted probabilities and observed overall survival rates, indicating the model's accuracy. Decision curve analysis (Fig. 8C) showed that the nomogram outperformed no-model and treat-all/none models across a range of threshold probabilities, suggesting its clinical utility. The ROC curve (Fig. 8D) further evaluated the discriminative ability of the nomogram, with AUC values of 0.669, 0.686, and 0.770 for 1-, 3-, and 5-year overall survival, respectively, indicating good predictive performance.

### Validation of SPI-related SPRG expression

**Fig. 9:**
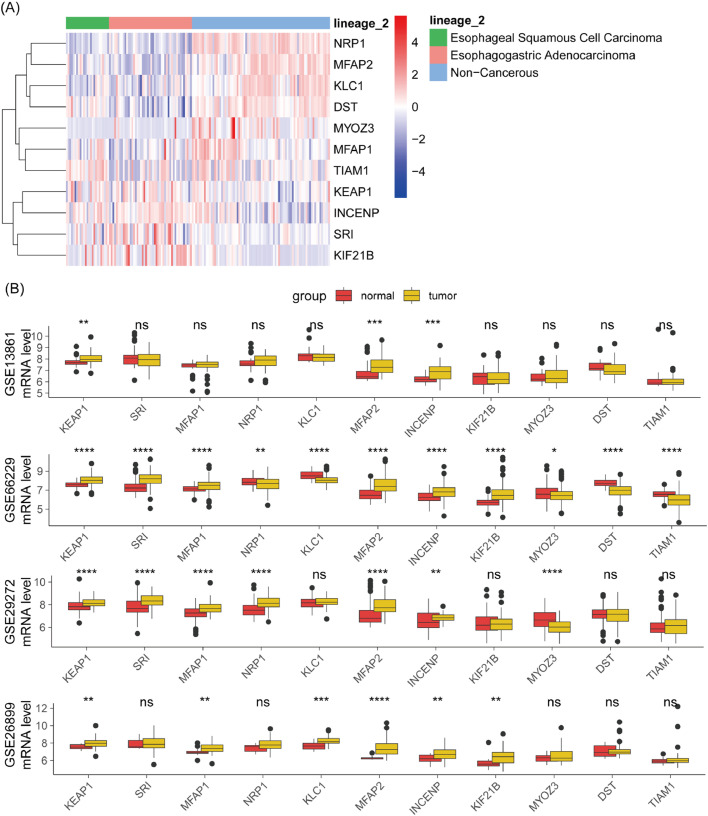
Validation of SPRG expression associated with risk score. (A) Heatmap of gene expression in esophageal-related cancers and non-cancerous cells from the CCLE database. Visualization of the expression levels of SPRGs in cancer versus normal cells. (B) Comparison of SPRG expression between tumor and normal tissues in the validation cohort. Box plots showing the expression levels of SPRGs in tumor versus adjacent normal tissues. Ns, Not significant; * *p* < 0.05; ** *p* < 0.01; *** *p* < 0.001, **** *p* < 0.0001.

The expression differences of SPI-related SPRGs between cancer and non-cancer cell lines were compared. Heatmap results (Fig. 9A) showed that NRP1, MFAP2, KLC1, DST, and MYOZ3 were generally downregulated in cancer cell lines, while KIF21B, SRI, INCENP, and KEAP1 were also downregulated. Further validation in four independent cohorts (Fig. 9B) showed that KEAP1, SRI, MFAP1, MFAP2, INCENP, and KIF21B were consistently upregulated in cancer tissues, while MYOZ3, DST, and TIAM1 were consistently downregulated. These results suggest that these genes may play important roles in cancer development. However, NRP1 and KLC1 did not show consistent expression patterns across different cohorts, indicating their more complex roles in cancer or influence by specific environmental factors.

### Biological functions of KLC1 in GC

**Fig. 10:**
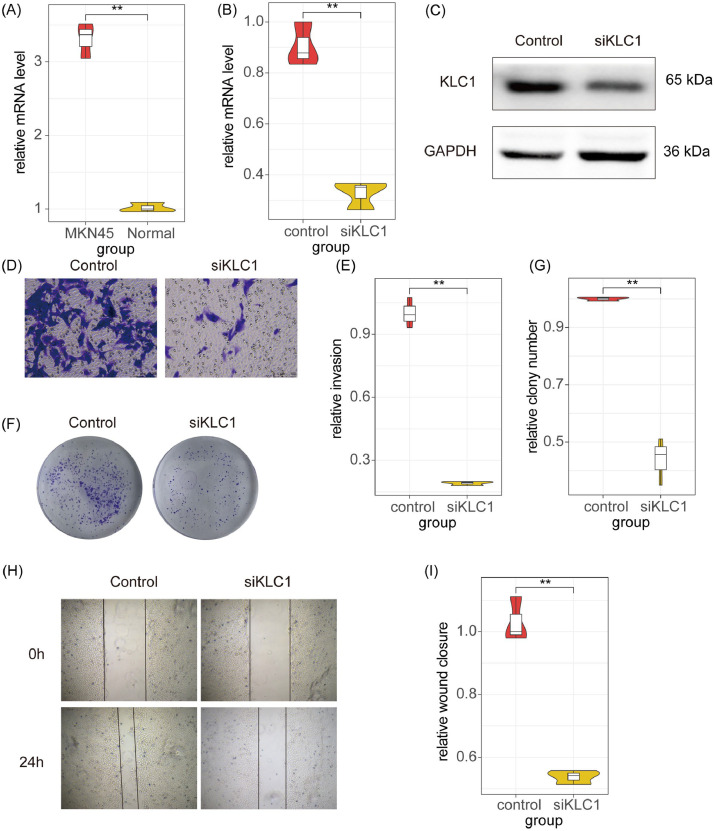
Biological function of KLC1 in GC cells. (A) Comparison of the mRNA level of KLC1 between the MKN45 and normal cell. (B) Comparison of the relative mRNA.level between the control and siKLC1 group. (C) Western blot analysis showing the protein expression levels of KLC1 and GAPDH (loading control) in control and siKLC1 groups. The protein level of KLC1 is reduced in the siKLC1 group. (D) Representative images of crystal violet-stained cell invasion assays for control and siKLC1 groups. Cells were stained blue, indicating invasive capacity. (E) Quantification of relative invasion rates between control and siKLC1 groups. The invasion rate is significantly lower in the siKLC1 group. (F) Colony formation assay showing the number of colonies formed by control and siKLC1 groups. Fewer colonies are observed in the siKLC1 group. (G) Quantification of relative colony numbers between control and siKLC1 groups. The number of colonies is significantly reduced in the siKLC1 group. (H) Wound healing assay images at 0 h and 24 h post-wounding for control and siKLC1 groups. The wound closure is slower in the siKLC1 group. (I) Quantification of relative wound closure between control and siKLC1 groups. The wound closure rate is significantly lower in the siKLC1 group. ** *p* < 0.01.

To investigate the functional role of KLC1 in GC, the authors first compared its expression levels between normal GES-1 and MKN45. As shown in Fig. 10A, KLC1 mRNA expression was significantly upregulated in MKN45 cells compared to GES-1 cells (*p* < 0.01). To explore the biological implications of this finding, the authors employed RNA interference technology to specifically knock down KLC1 expression in MKN45 cells. Both RT-qPCR (Fig. 10B) and Western blot analyses (Fig. 10C) confirmed efficient suppression of KLC1 at both mRNA and protein levels. Next, the authors evaluated the impact of KLC1 knockdown on the invasive capacity of MKN45 cells using Transwell invasion assays. It was observed that the relative frequency of invasive cells was significantly decreased in the KLC1-knockdown group compared to the control group (*p* < 0.01; Fig. 10D and E). The authors further examined the effects of KLC1 inhibition on cell proliferation and colony-forming ability using colony-formation assays. As illustrated in Fig. 10F and G, the number of colonies formed by MKN45 cells was markedly reduced upon KLC1 knockdown compared to the control group (*p* < 0.01). Additionally, wound-healing assays revealed that silencing KLC1 significantly impaired the migratory capability of MKN45 cells, as evidenced by delayed wound closure (Fig. 10H and I; *p* < 0.01). Collectively, these findings demonstrate that KLC1 plays a complex role in regulating the proliferation, motility, and invasiveness of GC cells.

## Discussion

This study successfully constructed a prognostic risk signature, SPI, based on SPRGs in GC and evaluated its potential for clinical application. Gastric cancer, as a leading cause of death worldwide, poses significant challenges due to its complex molecular heterogeneity and resistance to treatment. Developing new prognostic models to improve personalized treatment is therefore crucial. The analysis of TCGA-STAD data revealed that 182 SPRGs were significantly upregulated and 226 were downregulated in gastric cancer, indicating that SPRGs are closely associated with the molecular heterogeneity of the disease. Consensus clustering identified two molecular subtypes, with cluster 1 having significantly lower overall survival compared to cluster 2. This finding is in accordance with the results of previous studies, underscoring the significance of molecular subtypes in the prognosis of GC.^11^

Based on 11 selected SPRGs, the authors constructed a risk signature for GC, termed SPI, which effectively distinguished high-risk and low-risk patients in both the training and validation cohorts, with significant differences observed in their prognostic outcomes. However, the diagnostic accuracy of the SPI was relatively lower in the validation cohort, potentially attributable to multiple factors. First, the heterogeneity of technical platforms, such as RNA-seq from TCGA versus microarrays from GEO, introduces systematic biases in gene expression quantification.^12^ Second, systematic and hierarchical differences in the completeness of clinical metadata (i.e., non-omics features related to study endpoints) across datasets contribute to reduced cross-dataset predictive performance.^13^ Lastly, the spatiotemporal evolution of treatment strategies, such as the lack of immunotherapy data in datasets spanning 2008–2015, limits the model's ability to capture the impact of contemporary therapies.^14^ Therefore, future studies should validate the predictive capacity of the SPI through large-scale, multi-center, prospective cohort studies, incorporating more diverse populations and comprehensive clinical data to enhance its clinical applicability and generalizability.

Further exploration of the roles and mechanisms of SPI-related genes in cancer has revealed their critical involvement in cancer initiation, progression, and metastasis. For instance, KEAP1, a negative regulator of the Nrf2 signaling pathway, plays a pivotal role in cellular antioxidant responses and is associated with various types of cancer.^15^ Mutations in KEAP1 have been linked to immune therapy response and chemoresistance in cancer.^16^ In colitis-associated colorectal cancer, IKKβ drives inflammation and tumor progression, while KEAP1 promotes its degradation, exerting an anti-cancer effect.^17^ SRI is implicated in cell migration and invasion, with its upregulation associated with increased tumor invasiveness.^18^ Studies have shown that SRI regulates VEGFA/B expression via the PI3K pathway and negatively modulates pyroptosis by interacting with the NLRP3 inflammasome, thereby promoting hepatocellular carcinoma proliferation.^19^ Moreover, SRI functions by targeting EGFR signaling^18^ and Smad4.^20^

MFAP1 is involved in pre-mRNA processing and G2/M phase progression, while MFAP2 has been identified as a potential prognostic marker in multiple cancers.^21^ MFAP2 contributes to cancer progression and drug resistance by activating the FAK-AKT signaling pathway,^22^ Notch1 pathway,^23^ and Wnt/β-catenin signaling pathway.^24^ NRP1 has been extensively validated for its role in tumor drug resistance, particularly through the regulation of Yes-Associated Protein (YAP) and the PI3K/AKT signaling pathway.^25^ Furthermore, NRP1 has emerged as a potential biomarker in GC,^26^ where it regulates cell proliferation and apoptosis via the Wnt/β-catenin signaling pathway.^27^ KLC1 exhibits extensive alternative splicing, generating isoforms with distinct C-termini that bind to different proteins and play critical roles in cancer cells. Among these, KLC1-ALK represents a novel oncogenic fusion identified in lung cancer,^28^ while the KLC1-ROS1 fusion exerts oncogenic properties in glioma cells via specific activation of the JAK-STAT pathway.^29^ The in vitro experiments further validated the abnormal expression of KLC1 in GC and its biological functions in suppressing the proliferation, migration, and invasion of GC cells.

INCENP contributes to susceptibility to ER-negative breast cancer in European populations, and the arginine methylation of INCENP is involved in mitosis in cancer cells.^30^ Additionally, INCENP has been shown to be regulated by miR-1301–3p and contributes to the progression of esophageal squamous cell carcinoma.^31^ KIF21B, which plays a role in cell division, is associated with poor prognosis in colorectal and hepatocellular carcinomas.^32^^,^^33^ MYOZ3 is involved in muscle development and cell motility, and its function in tumor cells may relate to cellular migration and invasiveness. DST serves as a candidate tumor suppressor and potential biomarker in breast cancer, maintaining focal adhesion integrity, and promoting cell spreading and cell-matrix adhesion while preventing Zyxin accumulation, stabilizing LATS, and restricting YAP activation.^34^ Furthermore, DST regulates cisplatin resistance in colorectal cancer through the PI3K/AKT pathway. TIAM1 promotes cancer cell proliferation, invasion, and metastasis via multiple pathways, including AKT/mTOR signaling and ERK/STAT3 signaling.^35^ These findings highlight TIAM1′s multifaceted role in tumorigenesis and cancer progression.

Changes in the immune microenvironment significantly impact tumor progression and prognosis. In GC, the relationship between immune cell infiltration and SPRGs has received considerable attention. Studies have demonstrated that TIAM1 is essential for chemokine- and sphingosine-1-phosphate-induced Rac activation and subsequent cell migration, playing a role in regulating T-cell trafficking and trans-endothelial migration.^36^ Additionally, KIF21B has been shown to restrict microtubule growth, enabling rapid centrosome polarization in T-cells.^37^ Semaphorin 3 G acts as a key regulator of cancer immune responses by controlling the cytotoxicity of CD8+ *T* cells via NRP1, thereby suppressing tumor growth.^38^ Furthermore, NRP1 signaling can mediate the accumulation of Tregs within tumors.^39^ Moreover, KEAP1 regulates stem-like CD8+ *T*-cells and long-term T-cell immunity by inhibiting NRF2 activity.^40^ In the present study, the authors found that patients with high SPI exhibited increased infiltration of naive B cells, M2 macrophages, and activated NK cells, while M0 macrophages, resting NK cells, activated CD4 memory T-cells, and follicular helper T-cells were decreased. These findings suggest that SPRGs may modulate the composition of immune cells in the tumor microenvironment, thereby influencing disease progression and patient outcomes. ∼

However, the study has some limitations that warrant further investigation. First, the sample size may limit the generalizability of the results. Although the authors used data from multiple databases, including TCGA, GEO, and CCLE, these data primarily come from specific geographic regions and populations, which may not fully represent the global heterogeneity of gastric cancer. Future studies should validate the model in more diverse populations to improve external validity. Second, while the authors constructed SPI using various statistical methods and evaluated its association with clinical pathological features, more prospective clinical trials are needed to validate its predictive ability in actual treatment settings. Current analyses are based on retrospective data, which may be subject to selection bias. Additionally, although the authors assessed the relationship between SPI, drug sensitivity, and the immune microenvironment, the specific biological mechanisms of these genes in GC development remain to be explored. Future studies should combine laboratory research to uncover how these genes influence cellular signaling pathways and the microenvironment, thereby affecting GC prognosis.

## Conclusion

This study successfully constructed a prognostic risk signature based on SPRGs in gastric cancer, which is closely associated with clinical pathological features, drug sensitivity, and the immune landscape. SPI effectively distinguishes high-risk and low-risk patients and is related to tumor stage, lymph node metastasis, and tumor size. Immune landscape analysis revealed specific immune cell infiltration patterns in high-SPI patients. Additionally, the authors developed a nomogram model based on SPI and patient age, providing new insights for personalized treatment in gastric cancer.

## Abbreviations

GC, Gastric Cancer; SPRG, Supramolecular Polymer-Related Genes; TME, Tumor Microenvironment; PCA, Principal Component Analysis; KM, Kaplan-Meier; LASSO, Least Absolute Shrinkage and Selection Operator; ROC, Receiver Operating Characteristic; GO, Gene Ontology; TMB, Tumor Mutation Burden; GSEA, Gene Set Enrichment Analysis; DEG, Differentially Expressed Genes.

## Consent for publication

The author read and approved the final manuscript for publication.

## Ethics approval and consent to participate

No humans or animals were used in the present research.

## Informed consent

The authors declare that no patients were used in this study.

## Authors' contributions

Yan Liu, Hongyao Cui, Chuan Sun designed, extracted, analyzed and interpreted the data from databases. Yan Liu wrote the manuscript and made substantial contributions to the conception of the work and substantively revised it.

## Funding

None.

## Conflicts of interest

The authors declare no conflicts of interest.

## Data Availability

The data that support the findings of this study are available from the corresponding author upon reasonable request

## Appendix Supplementary materials

Image, application 1
