# Supplementary material for: The supramolecular polymer-related signature predicts prognosis and indicates immune microenvironment infiltration in gastric cancer
Source: Clinics (Sao Paulo). 2025 Apr 13;80:100641. doi: 10.1016/j.clinsp.2025.100641 (PMC12017930; doi:10.1016/j.clinsp.2025.100641)

**CLINICS-D-24-01385_ Supplementary Material**

**Supplementary Table S1** List of SUPRAMOLECULAR POLYMER-related genes.

| ABCC9 |
| --- |
| ABRA |
| ABRAXAS2 |
| ACKR2 |
| ACTA1 |
| ACTA2 |
| ACTB |
| ACTBL2 |
| ACTC1 |
| ACTG1 |
| ACTG2 |
| ACTL8 |
| ACTL9 |
| ACTN1 |
| ACTN2 |
| ACTN3 |
| ACTN4 |
| ADAMTS10 |
| ADAMTSL5 |
| ADORA2A |
| ADPRHL1 |
| AHNAK |
| AHNAK2 |
| AIF1 |
| AIF1L |
| AK1 |
| AKAP4 |
| AKNA |
| ALDOA |
| AMOT |
| ANK1 |
| ANK2 |
| ANK3 |
| ANKRD1 |
| ANKRD2 |
| ANKRD23 |
| ANXA1 |
| APC |
| APC2 |
| APPBP2 |
| ARF1 |
| ARFGEF2 |
| ARHGAP18 |
| ARHGAP4 |
| ARHGAP6 |
| ARHGEF2 |
| ARHGEF25 |
| ARL3 |
| ARL6 |
| ARPC3 |
| ASB2 |
| ASPM |
| ATAT1 |
| ATP2B4 |
| AURKA |
| AURKB |
| AURKC |
| AVIL |
| BAG2 |
| BAG3 |
| BAIAP2 |
| BBLN |
| BCAS3 |
| BCL10 |
| BEX4 |
| BFSP1 |
| BFSP2 |
| BICD1 |
| BIN1 |
| BIRC5 |
| BLOC1S6 |
| BMP10 |
| BOD1 |
| C10orf71 |
| CAB39 |
| CACNA1C |
| CACNA1D |
| CACNA1S |
| CALD1 |
| CALM1 |
| CALM2 |
| CALM3 |
| CAMSAP1 |
| CAMSAP2 |
| CAMSAP3 |
| CAPN3 |
| CAPN6 |
| CAPZB |
| CARMIL1 |
| CASP1 |
| CASP14 |
| CASQ1 |
| CASQ2 |
| CAV3 |
| CAVIN4 |
| CCDC181 |
| CCDC57 |
| CCDC66 |
| CCSAP |
| CCT2 |
| CCT3 |
| CCT4 |
| CCT5 |
| CCT6A |
| CCT7 |
| CCT8 |
| CD2AP |
| CDK1 |
| CDK2AP2 |
| CDK5 |
| CDK5R1 |
| CDK5RAP2 |
| CDK5RAP3 |
| CENPE |
| CENPJ |
| CEP162 |
| CEP170 |
| CEP170B |
| CEP295 |
| CEP57 |
| CEP57L1 |
| CFAP107 |
| CFAP126 |
| CFAP141 |
| CFAP144 |
| CFAP161 |
| CFAP20 |
| CFAP206 |
| CFAP210 |
| CFAP276 |
| CFAP45 |
| CFAP52 |
| CFAP53 |
| CFAP68 |
| CFAP77 |
| CFAP90 |
| CFAP95 |
| CFAP96 |
| CFL2 |
| CHMP1A |
| CHMP1B |
| CHMP2A |
| CHMP2B |
| CHMP3 |
| CHMP4A |
| CHMP4B |
| CHMP4BP1 |
| CHMP4C |
| CHMP5 |
| CHMP6 |
| CHMP7 |
| CIMAP1A |
| CIMAP1D |
| CIMIP2A |
| CIMIP2B |
| CIMIP2C |
| CKAP2 |
| CKAP5 |
| CLASP1 |
| CLASP2 |
| CLDN11 |
| CLIP1 |
| CLIP2 |
| CLIP3 |
| CLIP4 |
| CLMP |
| CLTC |
| CMYA5 |
| COBL |
| COL10A1 |
| COL11A1 |
| COL11A2 |
| COL1A1 |
| COL1A2 |
| COL27A1 |
| COL28A1 |
| COL2A1 |
| COL3A1 |
| COL4A1 |
| COL4A2 |
| COL4A3 |
| COL4A4 |
| COL4A5 |
| COL4A6 |
| COL5A1 |
| COL5A2 |
| COL5A3 |
| COL6A1 |
| COL6A3 |
| COL8A1 |
| CORO1A |
| CORO1B |
| CORO1C |
| COTL1 |
| CRHBP |
| CRYAB |
| CSNK1A1 |
| CSNK1D |
| CSPP1 |
| CSRP1 |
| CSRP2 |
| CSRP3 |
| CSTPP1 |
| CTNNB1 |
| CTPS1 |
| CTPS2 |
| CTTN |
| CUL3 |
| CYLD |
| CYP2A6 |
| DAG1 |
| DCDC1 |
| DCDC2 |
| DCDC2B |
| DCDC2C |
| DCTN1 |
| DCTN2 |
| DCTN4 |
| DCX |
| DCXR |
| DEK |
| DES |
| DIAPH1 |
| DIAPH2 |
| DIAPH3 |
| DISC1 |
| DLG1 |
| DLGAP2 |
| DMD |
| DMTN |
| DNAH1 |
| DNAH10 |
| DNAH11 |
| DNAH12 |
| DNAH14 |
| DNAH17 |
| DNAH2 |
| DNAH3 |
| DNAH5 |
| DNAH6 |
| DNAH7 |
| DNAH8 |
| DNAH9 |
| DNAI1 |
| DNAI2 |
| DNAJA3 |
| DNAJB4 |
| DNAJB6 |
| DNAL1 |
| DNAL4 |
| DNM1 |
| DNM1L |
| DNM2 |
| DNM3 |
| DPP9 |
| DPYSL2 |
| DPYSL3 |
| DSP |
| DST |
| DUSP21 |
| DUSP22 |
| DVL1 |
| DYNC1H1 |
| DYNC1I1 |
| DYNC1I2 |
| DYNC1LI1 |
| DYNC1LI2 |
| DYNC2H1 |
| DYNC2LI1 |
| DYNLL1 |
| DYNLL2 |
| DYNLRB1 |
| DYNLRB2 |
| DYNLT1 |
| DYNLT2 |
| DYNLT3 |
| EFCAB6 |
| EFEMP2 |
| EFHB |
| EFHC1 |
| EFHC2 |
| EIF3A |
| EIF6 |
| ELN |
| EMD |
| EML1 |
| EML2 |
| EML3 |
| EML4 |
| EML5 |
| EML6 |
| ENKD1 |
| ENKUR |
| ENO1 |
| EPPK1 |
| ESPN |
| EVPL |
| EZR |
| FAM110A |
| FAM110C |
| FAM161A |
| FAM161B |
| FAM83H |
| FBF1 |
| FBLN1 |
| FBLN5 |
| FBN1 |
| FBN2 |
| FBN3 |
| FBP2 |
| FBXL22 |
| FBXO22 |
| FBXO32 |
| FBXW11 |
| FERMT2 |
| FEZ1 |
| FGF13 |
| FHDC1 |
| FHL2 |
| FHL3 |
| FHL5 |
| FHOD3 |
| FIGN |
| FKBP1A |
| FKBP1B |
| FKBP4 |
| FKRP |
| FLACC1 |
| FLNA |
| FLNB |
| FLNC |
| FMN1 |
| FRG1 |
| FSD1 |
| FXR1 |
| FYN |
| GABARAP |
| GABARAPL1 |
| GABARAPL3 |
| GAS2 |
| GAS2L1 |
| GAS2L2 |
| GAS2L3 |
| GAS7 |
| GAS8 |
| GDPD2 |
| GFAP |
| GGPS1 |
| GJA1 |
| GJB6 |
| GLRX3 |
| GOLGA2 |
| GPER1 |
| GRAMD2B |
| GTSE1 |
| HABP4 |
| HAUS1 |
| HAUS2 |
| HAUS3 |
| HAUS4 |
| HAUS5 |
| HAUS6 |
| HAUS7 |
| HAUS8 |
| HCK |
| HDAC6 |
| HID1 |
| HLA-DRB1 |
| HNRNPU |
| HOMER1 |
| HOOK1 |
| HOOK2 |
| HOOK3 |
| HRC |
| HSPA8 |
| HSPB1 |
| HSPH1 |
| HTR2A |
| IDO1 |
| IFFO1 |
| IFFO2 |
| IFT70A |
| IFT70B |
| IGFN1 |
| ILK |
| INA |
| INCENP |
| INF2 |
| INO80 |
| INVS |
| IQGAP1 |
| ITGB1BP2 |
| JAKMIP1 |
| JAM3 |
| JPH1 |
| JPH2 |
| JUP |
| KAT2B |
| KATNA1 |
| KATNAL1 |
| KATNAL2 |
| KATNB1 |
| KCNA5 |
| KCNAB2 |
| KCNE1 |
| KCNJ8 |
| KCNN2 |
| KCTD6 |
| KEAP1 |
| KIF11 |
| KIF12 |
| KIF13A |
| KIF13B |
| KIF14 |
| KIF15 |
| KIF16B |
| KIF17 |
| KIF18A |
| KIF18B |
| KIF19 |
| KIF1A |
| KIF1B |
| KIF1C |
| KIF20A |
| KIF20B |
| KIF21A |
| KIF21B |
| KIF22 |
| KIF23 |
| KIF24 |
| KIF25 |
| KIF26A |
| KIF26B |
| KIF27 |
| KIF28P |
| KIF2A |
| KIF2B |
| KIF2C |
| KIF3A |
| KIF3B |
| KIF3C |
| KIF4A |
| KIF4B |
| KIF5A |
| KIF5B |
| KIF5C |
| KIF6 |
| KIF7 |
| KIF9 |
| KIFAP3 |
| KIFC1 |
| KIFC2 |
| KIFC3 |
| KLC1 |
| KLC2 |
| KLC3 |
| KLC4 |
| KLHL21 |
| KLHL22 |
| KLHL40 |
| KLHL41 |
| KNSTRN |
| KNTC1 |
| KPTN |
| KRT1 |
| KRT10 |
| KRT12 |
| KRT13 |
| KRT14 |
| KRT15 |
| KRT16 |
| KRT17 |
| KRT18 |
| KRT19 |
| KRT2 |
| KRT20 |
| KRT222 |
| KRT23 |
| KRT24 |
| KRT25 |
| KRT26 |
| KRT27 |
| KRT28 |
| KRT3 |
| KRT31 |
| KRT32 |
| KRT33A |
| KRT33B |
| KRT34 |
| KRT35 |
| KRT36 |
| KRT37 |
| KRT38 |
| KRT39 |
| KRT4 |
| KRT40 |
| KRT5 |
| KRT6A |
| KRT6B |
| KRT6C |
| KRT7 |
| KRT71 |
| KRT72 |
| KRT73 |
| KRT74 |
| KRT75 |
| KRT76 |
| KRT77 |
| KRT78 |
| KRT79 |
| KRT8 |
| KRT80 |
| KRT81 |
| KRT82 |
| KRT83 |
| KRT84 |
| KRT85 |
| KRT86 |
| KRT87P |
| KRT9 |
| KRTAP1-1 |
| KRTAP1-3 |
| KRTAP1-4 |
| KRTAP1-5 |
| KRTAP10-1 |
| KRTAP10-10 |
| KRTAP10-11 |
| KRTAP10-12 |
| KRTAP10-2 |
| KRTAP10-3 |
| KRTAP10-4 |
| KRTAP10-5 |
| KRTAP10-6 |
| KRTAP10-7 |
| KRTAP10-8 |
| KRTAP10-9 |
| KRTAP11-1 |
| KRTAP12-1 |
| KRTAP12-2 |
| KRTAP12-3 |
| KRTAP12-4 |
| KRTAP13-1 |
| KRTAP13-2 |
| KRTAP13-3 |
| KRTAP13-4 |
| KRTAP15-1 |
| KRTAP16-1 |
| KRTAP17-1 |
| KRTAP19-1 |
| KRTAP19-2 |
| KRTAP19-3 |
| KRTAP19-4 |
| KRTAP19-5 |
| KRTAP19-6 |
| KRTAP19-7 |
| KRTAP19-8 |
| KRTAP2-1 |
| KRTAP2-3 |
| KRTAP2-4 |
| KRTAP20-1 |
| KRTAP20-2 |
| KRTAP20-3 |
| KRTAP20-4 |
| KRTAP21-1 |
| KRTAP21-2 |
| KRTAP21-3 |
| KRTAP22-1 |
| KRTAP22-2 |
| KRTAP23-1 |
| KRTAP24-1 |
| KRTAP25-1 |
| KRTAP26-1 |
| KRTAP27-1 |
| KRTAP29-1 |
| KRTAP3-1 |
| KRTAP3-2 |
| KRTAP3-3 |
| KRTAP4-1 |
| KRTAP4-11 |
| KRTAP4-12 |
| KRTAP4-16 |
| KRTAP4-2 |
| KRTAP4-3 |
| KRTAP4-4 |
| KRTAP4-5 |
| KRTAP4-6 |
| KRTAP4-8 |
| KRTAP4-9 |
| KRTAP5-1 |
| KRTAP5-10 |
| KRTAP5-11 |
| KRTAP5-2 |
| KRTAP5-3 |
| KRTAP5-4 |
| KRTAP5-5 |
| KRTAP5-6 |
| KRTAP5-7 |
| KRTAP5-8 |
| KRTAP5-9 |
| KRTAP6-1 |
| KRTAP6-2 |
| KRTAP6-3 |
| KRTAP7-1 |
| KRTAP8-1 |
| KRTAP9-1 |
| KRTAP9-2 |
| KRTAP9-3 |
| KRTAP9-4 |
| KRTAP9-6 |
| KRTAP9-7 |
| KRTAP9-8 |
| KRTAP9-9 |
| KY |
| LCP1 |
| LDB3 |
| LDLRAP1 |
| LMAN1 |
| LMNA |
| LMNB1 |
| LMNB2 |
| LMNTD1 |
| LMNTD2 |
| LMOD1 |
| LMOD2 |
| LMOD3 |
| LRPPRC |
| LRRC10 |
| LRRC27 |
| LRRC39 |
| LRRC49 |
| LTBP1 |
| LTBP4 |
| LUM |
| LZTS2 |
| MACF1 |
| MAP10 |
| MAP1A |
| MAP1B |
| MAP1LC3A |
| MAP1LC3B |
| MAP1LC3B2 |
| MAP1LC3C |
| MAP1S |
| MAP2 |
| MAP2K2 |
| MAP3K11 |
| MAP4 |
| MAP6 |
| MAP6D1 |
| MAP7 |
| MAP9 |
| MAPRE1 |
| MAPRE2 |
| MAPRE3 |
| MAPT |
| MARK2 |
| MATCAP1 |
| MDM1 |
| MEFV |
| MFAP1 |
| MFAP2 |
| MFAP4 |
| MFAP5 |
| MICAL1 |
| MICAL2 |
| MID1 |
| MID1IP1 |
| MID2 |
| MISP |
| MMP2 |
| MNS1 |
| MT3 |
| MTA1 |
| MTCL1 |
| MTCL2 |
| MTM1 |
| MTMR12 |
| MTUS1 |
| MTUS2 |
| MX1 |
| MX2 |
| MYBPC1 |
| MYBPC2 |
| MYBPC3 |
| MYBPH |
| MYBPHL |
| MYH1 |
| MYH10 |
| MYH11 |
| MYH13 |
| MYH14 |
| MYH15 |
| MYH2 |
| MYH3 |
| MYH4 |
| MYH6 |
| MYH7 |
| MYH7B |
| MYH8 |
| MYH9 |
| MYL1 |
| MYL11 |
| MYL12B |
| MYL2 |
| MYL3 |
| MYL4 |
| MYL5 |
| MYL6B |
| MYL7 |
| MYL9 |
| MYLK2 |
| MYO18A |
| MYO18B |
| MYO1A |
| MYO1B |
| MYO1C |
| MYO3A |
| MYO5A |
| MYO6 |
| MYO9A |
| MYO9B |
| MYOD1 |
| MYOM1 |
| MYOM2 |
| MYOM3 |
| MYOT |
| MYOZ1 |
| MYOZ2 |
| MYOZ3 |
| MYPN |
| MYZAP |
| NARF |
| NAV1 |
| NAV3 |
| NBR1 |
| NCKAP1 |
| NCKAP5 |
| NCKAP5L |
| NCKIPSD |
| NDE1 |
| NDEL1 |
| NDRG1 |
| NEB |
| NEBL |
| NEFH |
| NEFL |
| NEFM |
| NEK2 |
| NEK6 |
| NEK7 |
| NES |
| NEXN |
| NICN1 |
| NIN |
| NINL |
| NME7 |
| NOS1AP |
| NPNT |
| NRAP |
| NRP1 |
| NUDC |
| NUMA1 |
| NUSAP1 |
| OBSCN |
| OBSL1 |
| ODAM |
| ODF1 |
| ODF2 |
| OPA1 |
| PACRG |
| PAFAH1B1 |
| PAK1 |
| PALLD |
| PARP4 |
| PARVA |
| PARVB |
| PAWR |
| PBXIP1 |
| PCNT |
| PDE4B |
| PDE4DIP |
| PDLIM1 |
| PDLIM2 |
| PDLIM3 |
| PDLIM4 |
| PDLIM5 |
| PDLIM7 |
| PGM5 |
| PIERCE1 |
| PIERCE2 |
| PKP1 |
| PKP2 |
| PLEC |
| PLK1 |
| PLS1 |
| PLS3 |
| PNN |
| POF1B |
| POLB |
| POLR2M |
| POTEE |
| POTEF |
| POTEI |
| POTEJ |
| POTEKP |
| PPL |
| PPP1R12A |
| PPP1R12B |
| PPP2R5A |
| PPP3CA |
| PPP3CB |
| PRC1 |
| PRICKLE4 |
| PRKD1 |
| PRPH |
| PSMA6 |
| PSRC1 |
| PSTPIP1 |
| PSTPIP2 |
| PTPN20 |
| PVALEF |
| PYCARD |
| PYROXD1 |
| RAB11A |
| RAB3D |
| RAC1 |
| RAC2 |
| RAC3 |
| RACGAP1 |
| RADIL |
| RASSF1 |
| RASSF3 |
| RASSF5 |
| RCC2 |
| RCSD1 |
| REEP1 |
| REEP2 |
| REEP3 |
| REEP4 |
| REM1 |
| RGS14 |
| RHOQ |
| RIBC1 |
| RIBC2 |
| RMDN1 |
| RMDN2 |
| RMDN3 |
| RNF4 |
| ROR2 |
| RP1 |
| RP1L1 |
| RPGRIP1L |
| RPL15 |
| RSPH1 |
| RTN2 |
| RUSC1 |
| RYR1 |
| RYR2 |
| RYR3 |
| SAA1 |
| SARM1 |
| SAXO1 |
| SAXO2 |
| SCN1A |
| SCN3B |
| SCN5A |
| SCN8A |
| SCO1 |
| SCO2 |
| SCTR |
| SCYGR1 |
| SCYGR10 |
| SCYGR2 |
| SCYGR3 |
| SCYGR4 |
| SCYGR5 |
| SCYGR6 |
| SCYGR7 |
| SCYGR8 |
| SCYGR9 |
| SDC4 |
| SELENOS |
| SEPTIN9 |
| SERP1 |
| SH2B2 |
| SHANK2 |
| SHROOM1 |
| SHROOM2 |
| SHROOM3 |
| SHROOM4 |
| SHTN1 |
| SIMC1 |
| SIRT2 |
| SKA1 |
| SKA2 |
| SKA3 |
| SLAIN1 |
| SLAIN2 |
| SLC1A4 |
| SLC2A1 |
| SLC4A1 |
| SLC8A1 |
| SLMAP |
| SMN1 |
| SMN2 |
| SMPX |
| SMTN |
| SMTNL1 |
| SMTNL2 |
| SNCA |
| SNPH |
| SNTB2 |
| SORBS2 |
| SPACA9 |
| SPAG17 |
| SPAG5 |
| SPAG6 |
| SPAG8 |
| SPAST |
| SPECC1 |
| SPECC1L |
| SPEF1 |
| SPMIP10 |
| SPMIP11 |
| SPMIP6 |
| SPMIP8 |
| SPMIP9 |
| SPRY2 |
| SPTBN1 |
| SPTBN4 |
| SQSTM1 |
| SRC |
| SRI |
| SRPRB |
| SSNA1 |
| STAU2 |
| STIM1 |
| STK11 |
| STMN1 |
| STUB1 |
| STYXL2 |
| SVIL |
| SYBU |
| SYNC |
| SYNE1 |
| SYNE2 |
| SYNJ1 |
| SYNM |
| SYNPO |
| SYNPO2 |
| SYNPO2L |
| TBCA |
| TBCB |
| TBCC |
| TBCD |
| TBCE |
| TCAP |
| TCHP |
| TCP1 |
| TCP11L1 |
| TEK |
| TEKT1 |
| TEKT2 |
| TEKT3 |
| TEKT4 |
| TEKT5 |
| TEKTIP1 |
| TEKTL1 |
| TFPT |
| THSD4 |
| TIAM1 |
| TLK2 |
| TMEM214 |
| TMEM232 |
| TMOD1 |
| TMOD2 |
| TMOD3 |
| TMOD4 |
| TNK2 |
| TNNC1 |
| TNNC2 |
| TNNI1 |
| TNNI2 |
| TNNI3 |
| TNNT1 |
| TNNT2 |
| TNNT3 |
| TOGARAM1 |
| TOGARAM2 |
| TPGS1 |
| TPGS2 |
| TPM1 |
| TPM2 |
| TPM3 |
| TPM4 |
| TPPP |
| TPPP2 |
| TPPP3 |
| TPT1 |
| TPX2 |
| TRIM32 |
| TRIM54 |
| TRIM55 |
| TRIM63 |
| TRPV4 |
| TSC1 |
| TTL |
| TTLL1 |
| TTLL11 |
| TTLL13 |
| TTLL3 |
| TTLL4 |
| TTLL5 |
| TTLL6 |
| TTLL7 |
| TTLL8 |
| TTLL9 |
| TTN |
| TUBA1A |
| TUBA1B |
| TUBA1C |
| TUBA3C |
| TUBA3D |
| TUBA3E |
| TUBA4A |
| TUBA4B |
| TUBA8 |
| TUBAL3 |
| TUBB |
| TUBB1 |
| TUBB2A |
| TUBB2B |
| TUBB3 |
| TUBB4A |
| TUBB4B |
| TUBB6 |
| TUBB8 |
| TUBB8B |
| TUBD1 |
| TUBE1 |
| TUBG1 |
| TUBG2 |
| TUBGCP2 |
| TUBGCP3 |
| TUBGCP4 |
| TUBGCP5 |
| TUBGCP6 |
| TWF1 |
| TWF2 |
| UNC45B |
| UPP2 |
| VCL |
| VIM |
| VMAC |
| VPS18 |
| WAS |
| WDR47 |
| WHAMM |
| WHRN |
| WIPF1 |
| XIRP2 |
| YES1 |
| ZNF207 |
| ZNF804A |
| ZW10 |
| ZWILCH |

**Supplementary** **Table S2** SPRGs associated with the overall survival of gastric cancer.

| **Gene** | **HR** | **Lower95** | **Upper95** | **p** |
| --- | --- | --- | --- | --- |
| LMOD1 | 1.08 | 1.00 | 1.17 | 0.0489 |
| CASQ2 | 1.13 | 1.00 | 1.27 | 0.0487 |
| KRT86 | 1.53 | 1.00 | 2.32 | 0.0481 |
| CRYAB | 1.13 | 1.00 | 1.27 | 0.0479 |
| TOGARAM1 | 1.47 | 1.00 | 2.14 | 0.0477 |
| CYP2A6 | 1.61 | 1.01 | 2.57 | 0.0473 |
| PARVA | 1.22 | 1.00 | 1.50 | 0.0466 |
| FAM83H | 0.86 | 0.73 | 1.00 | 0.0462 |
| NUDC | 0.72 | 0.52 | 0.99 | 0.0462 |
| KLC1 | 1.92 | 1.01 | 3.63 | 0.0455 |
| DNAH6 | 0.51 | 0.27 | 0.98 | 0.0445 |
| KRT2 | 1.66 | 1.01 | 2.71 | 0.0440 |
| CEP57 | 0.66 | 0.44 | 0.99 | 0.0439 |
| KRT15 | 0.82 | 0.68 | 0.99 | 0.0439 |
| COL5A1 | 1.15 | 1.00 | 1.32 | 0.0438 |
| PKP2 | 0.82 | 0.68 | 0.99 | 0.0422 |
| CMYA5 | 1.81 | 1.02 | 3.20 | 0.0422 |
| KIF5C | 3.01 | 1.05 | 8.66 | 0.0410 |
| KIF21B | 0.74 | 0.56 | 0.99 | 0.0408 |
| KEAP1 | 0.69 | 0.48 | 0.98 | 0.0393 |
| LTBP1 | 1.16 | 1.01 | 1.34 | 0.0385 |
| SNCA | 1.47 | 1.02 | 2.12 | 0.0384 |
| CCDC181 | 1.63 | 1.03 | 2.59 | 0.0381 |
| SYNPO | 1.19 | 1.01 | 1.41 | 0.0375 |
| FLNA | 1.12 | 1.01 | 1.24 | 0.0374 |
| XIRP2 | 14.87 | 1.17 | 188.41 | 0.0372 |
| COL1A1 | 1.13 | 1.01 | 1.26 | 0.0368 |
| MFAP1 | 0.59 | 0.36 | 0.97 | 0.0367 |
| DPP9 | 0.63 | 0.41 | 0.97 | 0.0367 |
| MYH10 | 1.20 | 1.01 | 1.42 | 0.0366 |
| SRI | 1.30 | 1.02 | 1.66 | 0.0358 |
| TPM2 | 1.11 | 1.01 | 1.22 | 0.0357 |
| MFAP5 | 1.14 | 1.01 | 1.29 | 0.0348 |
| WHRN | 0.77 | 0.61 | 0.98 | 0.0343 |
| COL4A2 | 1.21 | 1.01 | 1.44 | 0.0342 |
| ACTA1 | 1.30 | 1.02 | 1.65 | 0.0336 |
| CKAP2 | 0.79 | 0.64 | 0.98 | 0.0335 |
| NAV1 | 1.37 | 1.03 | 1.83 | 0.0332 |
| LTBP4 | 1.20 | 1.01 | 1.42 | 0.0331 |
| CRHBP | 2.31 | 1.07 | 4.98 | 0.0329 |
| MARK2 | 0.67 | 0.46 | 0.97 | 0.0329 |
| CDK5 | 0.73 | 0.54 | 0.97 | 0.0324 |
| ACKR2 | 1.49 | 1.03 | 2.14 | 0.0320 |
| TEK | 1.30 | 1.03 | 1.64 | 0.0298 |
| POF1B | 0.87 | 0.77 | 0.99 | 0.0297 |
| COL10A1 | 1.11 | 1.01 | 1.22 | 0.0294 |
| TMOD2 | 1.40 | 1.03 | 1.90 | 0.0292 |
| TMOD1 | 1.19 | 1.02 | 1.38 | 0.0291 |
| MYH6 | 6.06 | 1.21 | 30.29 | 0.0282 |
| FEZ1 | 1.41 | 1.04 | 1.91 | 0.0281 |
| COL5A2 | 1.18 | 1.02 | 1.37 | 0.0281 |
| POTEJ | 4.27 | 1.17 | 15.58 | 0.0278 |
| MYL4 | 2.00 | 1.08 | 3.68 | 0.0264 |
| CHMP4C | 0.83 | 0.70 | 0.98 | 0.0262 |
| KIF5A | 1.72 | 1.07 | 2.78 | 0.0258 |
| COL4A1 | 1.24 | 1.03 | 1.50 | 0.0258 |
| PDLIM3 | 1.14 | 1.02 | 1.27 | 0.0258 |
| KIF3C | 1.29 | 1.03 | 1.61 | 0.0254 |
| GABARAPL1 | 1.28 | 1.03 | 1.59 | 0.0253 |
| KIF9 | 0.61 | 0.40 | 0.94 | 0.0247 |
| EML5 | 5.55 | 1.26 | 24.43 | 0.0236 |
| TTN | 6.42 | 1.28 | 32.09 | 0.0235 |
| COL1A2 | 1.15 | 1.02 | 1.30 | 0.0233 |
| GGPS1 | 1.68 | 1.07 | 2.62 | 0.0232 |
| MAP2 | 1.33 | 1.04 | 1.70 | 0.0227 |
| CFL2 | 1.16 | 1.02 | 1.32 | 0.0226 |
| TIAM1 | 1.38 | 1.05 | 1.82 | 0.0222 |
| EFEMP2 | 1.23 | 1.03 | 1.47 | 0.0206 |
| MYL9 | 1.11 | 1.02 | 1.20 | 0.0196 |
| TPPP2 | 9.81 | 1.45 | 66.38 | 0.0193 |
| COL3A1 | 1.15 | 1.02 | 1.29 | 0.0191 |
| SPAG6 | 2.10 | 1.13 | 3.89 | 0.0190 |
| KIF28P | 3.69 | 1.24 | 10.96 | 0.0186 |
| COL4A5 | 1.23 | 1.04 | 1.47 | 0.0185 |
| FBLN1 | 1.12 | 1.02 | 1.23 | 0.0180 |
| ACTA2 | 1.13 | 1.02 | 1.24 | 0.0178 |
| KIF24 | 0.70 | 0.52 | 0.94 | 0.0173 |
| MTUS2 | 2.31 | 1.16 | 4.59 | 0.0172 |
| MAP1B | 1.18 | 1.03 | 1.35 | 0.0168 |
| POTEI | 18.44 | 1.71 | 199.50 | 0.0164 |
| MAP10 | 1.40 | 1.06 | 1.84 | 0.0158 |
| ADAMTS10 | 1.37 | 1.06 | 1.76 | 0.0157 |
| LUM | 1.17 | 1.03 | 1.34 | 0.0155 |
| DYNLRB2 | 3.72 | 1.30 | 10.65 | 0.0144 |
| CALD1 | 1.14 | 1.03 | 1.27 | 0.0138 |
| LMNB2 | 0.75 | 0.60 | 0.94 | 0.0137 |
| TUBB4A | 1.33 | 1.06 | 1.67 | 0.0135 |
| CLIP3 | 1.18 | 1.04 | 1.35 | 0.0135 |
| TUBG2 | 1.39 | 1.07 | 1.81 | 0.0133 |
| TUBA1A | 1.18 | 1.04 | 1.35 | 0.0132 |
| FBN2 | 1.57 | 1.10 | 2.24 | 0.0130 |
| C10orf71 | 33.78 | 2.19 | 522.28 | 0.0118 |
| TEKT2 | 1.78 | 1.14 | 2.78 | 0.0116 |
| DNAH8 | 3.82 | 1.35 | 10.77 | 0.0114 |
| DYNC1I1 | 1.36 | 1.07 | 1.73 | 0.0113 |
| ABCC9 | 1.26 | 1.05 | 1.50 | 0.0110 |
| ROR2 | 1.21 | 1.05 | 1.41 | 0.0108 |
| ACTN3 | 2.02 | 1.18 | 3.46 | 0.0102 |
| MFAP2 | 1.20 | 1.04 | 1.38 | 0.0101 |
| TUBB6 | 1.23 | 1.05 | 1.45 | 0.0098 |
| MAP6 | 1.34 | 1.07 | 1.67 | 0.0097 |
| DNM3 | 1.71 | 1.14 | 2.56 | 0.0094 |
| DST | 1.27 | 1.06 | 1.52 | 0.0089 |
| KIF17 | 2.04 | 1.20 | 3.47 | 0.0084 |
| DPYSL3 | 1.15 | 1.04 | 1.27 | 0.0083 |
| COL8A1 | 1.18 | 1.04 | 1.33 | 0.0082 |
| INCENP | 0.70 | 0.54 | 0.91 | 0.0077 |
| JAM3 | 1.25 | 1.06 | 1.47 | 0.0074 |
| KRT81 | 1.25 | 1.06 | 1.48 | 0.0073 |
| KIFAP3 | 1.46 | 1.11 | 1.93 | 0.0072 |
| VIM | 1.30 | 1.08 | 1.57 | 0.0067 |
| ELN | 1.17 | 1.05 | 1.32 | 0.0066 |
| DISC1 | 2.21 | 1.25 | 3.91 | 0.0063 |
| KCNJ8 | 1.33 | 1.09 | 1.63 | 0.0058 |
| AK1 | 0.67 | 0.51 | 0.89 | 0.0054 |
| CLIP4 | 1.38 | 1.10 | 1.72 | 0.0047 |
| KRT72 | 3.06 | 1.42 | 6.62 | 0.0045 |
| FERMT2 | 1.21 | 1.06 | 1.39 | 0.0044 |
| IGFN1 | 1.80 | 1.20 | 2.70 | 0.0044 |
| GJA1 | 1.24 | 1.07 | 1.44 | 0.0038 |
| NAV3 | 1.99 | 1.25 | 3.15 | 0.0035 |
| FBLN5 | 1.24 | 1.07 | 1.43 | 0.0032 |
| TUBB8 | 13.24 | 2.38 | 73.62 | 0.0032 |
| FBN1 | 1.24 | 1.08 | 1.44 | 0.0031 |
| MID2 | 1.37 | 1.11 | 1.69 | 0.0030 |
| BEX4 | 1.25 | 1.08 | 1.44 | 0.0024 |
| MYOZ3 | 2.62 | 1.41 | 4.88 | 0.0023 |
| MYO5A | 1.50 | 1.17 | 1.93 | 0.0016 |
| PRKD1 | 1.57 | 1.20 | 2.04 | 0.0009 |
| DLGAP2 | 18.58 | 3.32 | 103.87 | 0.0009 |
| GFAP | 19.63 | 3.41 | 112.91 | 0.0009 |
| DCDC2C | 10.91 | 2.77 | 42.99 | 0.0006 |
| ANKRD1 | 1.41 | 1.16 | 1.72 | 0.0006 |
| RADIL | 2.15 | 1.39 | 3.31 | 0.0006 |
| NRP1 | 1.56 | 1.23 | 1.97 | 0.0002 |
| CLDN11 | 1.43 | 1.19 | 1.73 | 0.0002 |

**Supplementary** **Table S3** Lasso identified 11 SPRGs with non-zero coefficients.

| **Gene Name** | **Full Name** | **NCBI Gene ID** |
| --- | --- | --- |
| KEAP1 | Kelch-like ECH-associated protein 1 | 9817 |
| SRI | sorcin | 6717 |
| MFAP1 | Microfibrillar-associated protein 1 | 4236 |
| NRP1 | Neuropilin 1 | 8829 |
| KLC1 | Kinesin light chain cube 1 | 3831 |
| MFAP2 | Microfibrillar-associated protein 2 | 4237 |
| INCENP | Inner centromere protein | 3619 |
| KIF21B | Kinesin family member 21B | 23046 |
| MYOZ3 | Myozenin 3 | 91977 |
| DST | [dystonin](https://www.ncbi.nlm.nih.gov/gene/667) | 667 |
| TIAM1 | TIAM Rac1 associated GEF 1 | 7074 |

**Supplementary** **Table S4** Summary of the clinicopathological features of the included cohorts used in this study.

| **Variables** | **TCGA-COAD** | | | **GSE26253** | | | **GSE84437** | | |
| --- | --- | --- | --- | --- | --- | --- | --- | --- | --- |
|  | **High** | **Low** | **p** | **High** | **Low** | **p** | **High** | **Low** | **p** |
| Age | 64.26 ± 10.43 | 65.08 ± 10.44 | 0.466 |  |  |  | 59.42±12.37 | 60.75±10.66 | 0.232 |
| **Outcomes** |  |  | **<0.0001** |  |  | **0.04** |  |  | **0.007** |
| Dead | 89 | 52 |  | 94 | 83 |  | 122 | 87 |  |
| Alive | 82 | 118 |  | 109 | 146 |  | 101 | 123 |  |
| M stage |  |  | 0.27 |  |  |  |  |  |  |
| M0 | 157 | 162 |  |  |  |  |  |  |  |
| M1 | 14 | 8 |  |  |  |  |  |  |  |
| N stage |  |  | 0.078 |  |  |  |  |  | 0.058 |
| N0 | 42 | 62 |  |  |  |  | 37 | 43 |  |
| N1 | 51 | 44 |  |  |  |  | 90 | 98 |  |
| N2 | 41 | 28 |  |  |  |  | 81 | 51 |  |
| N3 | 37 | 36 |  |  |  |  | 15 | 18 |  |
| T stage |  |  | 0.056 |  |  |  |  |  | 0.097 |
| T1 | 3 | 12 |  |  |  |  | 4 | 7 |  |
| T2 | 35 | 38 |  |  |  |  | 15 | 23 |  |
| T3 | 88 | 74 |  |  |  |  | 42 | 50 |  |
| T4 | 45 | 45 |  |  |  |  | 162 | 130 |  |
| Gender |  |  | 0.498 |  |  |  |  |  | 0.836 |
| female | 64 | 57 |  |  |  |  | 72 | 65 |  |
| male | 107 | 113 |  |  |  |  | 151 | 145 |  |
| Stage |  |  | 0.275 |  |  | 0.135 |  |  |  |
| I | 19 | 31 |  | 28 | 40 |  |  |  |  |
| II | 55 | 55 |  | 71 | 96 |  |  |  |  |
| III | 80 | 68 |  | 66 | 64 |  |  |  |  |
| IV | 17 | 16 |  | 38 | 29 |  |  |  |  |

**Supplementary Table S5** Univariate and multivariate cox analysis identified independent prognostic factors for GC.

| **Characteristics** | **HR [95% CI]** | **p-value** | **HR [95% CI]** | **p-value** |
| --- | --- | --- | --- | --- |
| Riskscore | 4.4 [2.7‒7.1] | 2.4e-09 | 4.73 [2.82, 7.93] | <0.000001 |
| Age | 1 [1‒1] | 0.042 | 1.03 [1.01, 1.05] | 0.000981 |
| T_stage | 1.3 [1.1‒1.6] | 0.012 | 1.34 [0.88, 2.03] | 0.174185 |
| N_stage | 1.4 [1.2‒1.6] | 5.2e-05 | 1.16 [0.86, 1.56] | 0.323441 |
| M_stage | 2 [1.1‒3.6] | 0.023 | 1.14 [0.92, 1.43] | 0.237857 |
| Gender | 1.3 [0.92‒1.9] | 0.13 | 1.51 [0.71, 3.22] | 0.289294 |
| Stage | 1.6 [1.3‒2] | 1e-05 | 1.27 [0.88, 1.84] | 0.205604 |

**Supplementary** **Figure S1** Consensus clustering of the TCGA-STAD cohort based on SPRGs. (A) Consensus Cumulative Distribution Function (CDF) Plot. The curves represent different numbers of clusters (k), with the step change in the CDF curve at k=3 suggesting a potential optimal cluster number. (B) Delta area plot illustrates the relative change in the area under the CDF curve for varying values of k. (C) Consensus Matrix for k=2: The heatmap displays the consensus matrix when k is set to 2, indicating the stability of clustering assignments.


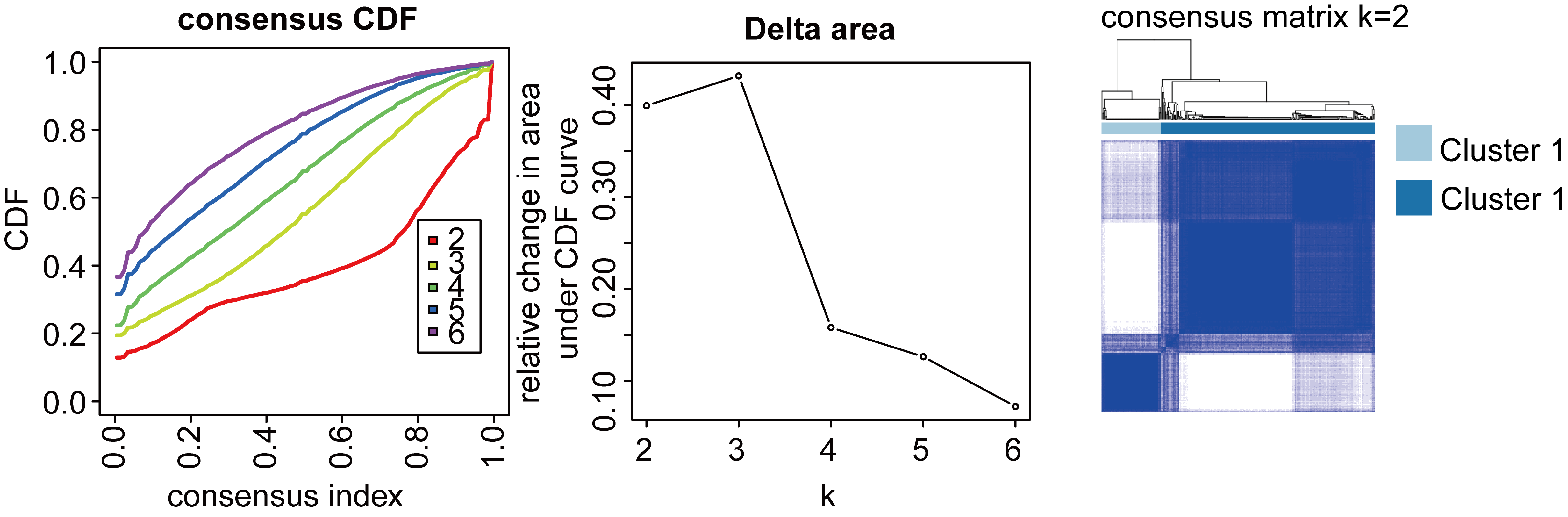


**Supplementary** **Figure S2** LASSO regression analysis for feature selection in gastric cancer prognosis. (A) Coefficient path plot. This plot illustrates the shrinkage of regression coefficients as the regularization parameter, lambda (λ), increases from left to right on the x-axis. (B) Partial likelihood deviance plot. It shows the partial likelihood deviance for various values of lambda, which helps in selecting an optimal lambda value. (C) Distribution of the coefficients associated with SPI-related genes.


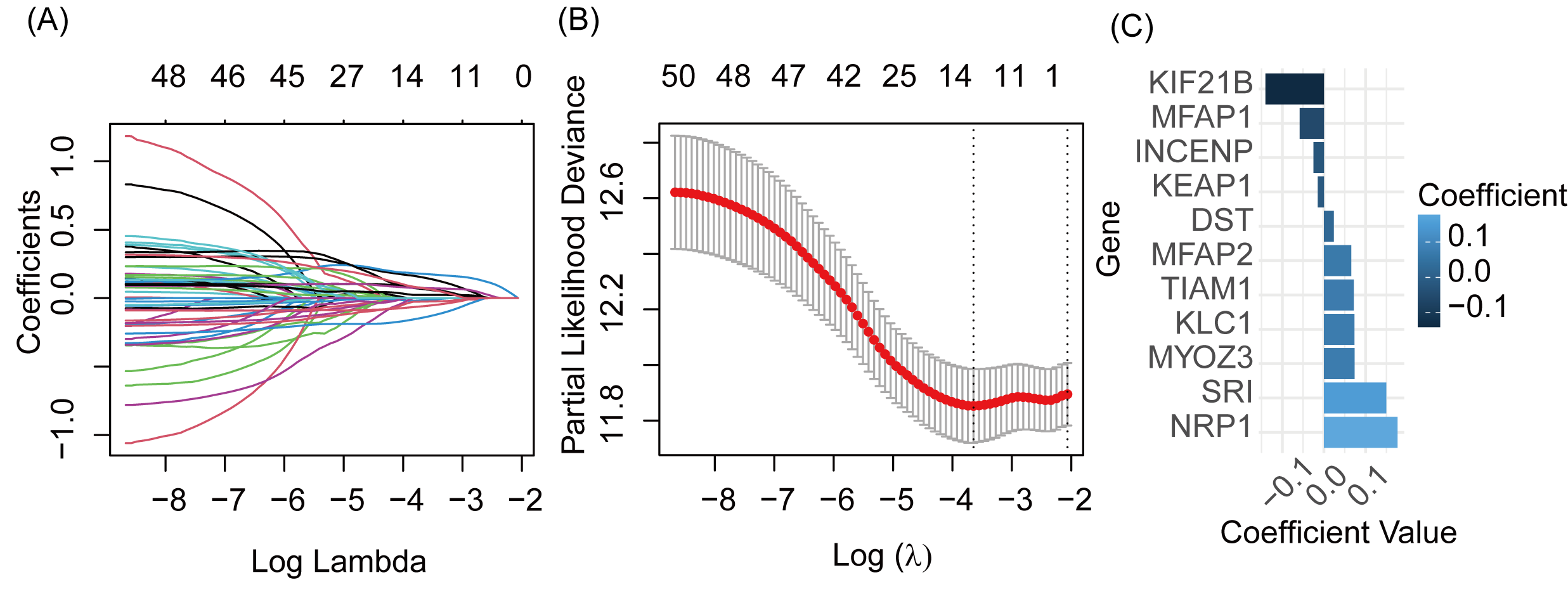


**Supplementary** **Figure S3** Mutational landscape analysis across different groups in gastric cancer. (A‒F) Proportion of mutation types for ACVR2A, PCDH15, NRXN1, SPEN, VCAN, and ADGRB3. Pie charts depicting the distribution of mutation types for each gene across the studied groups.


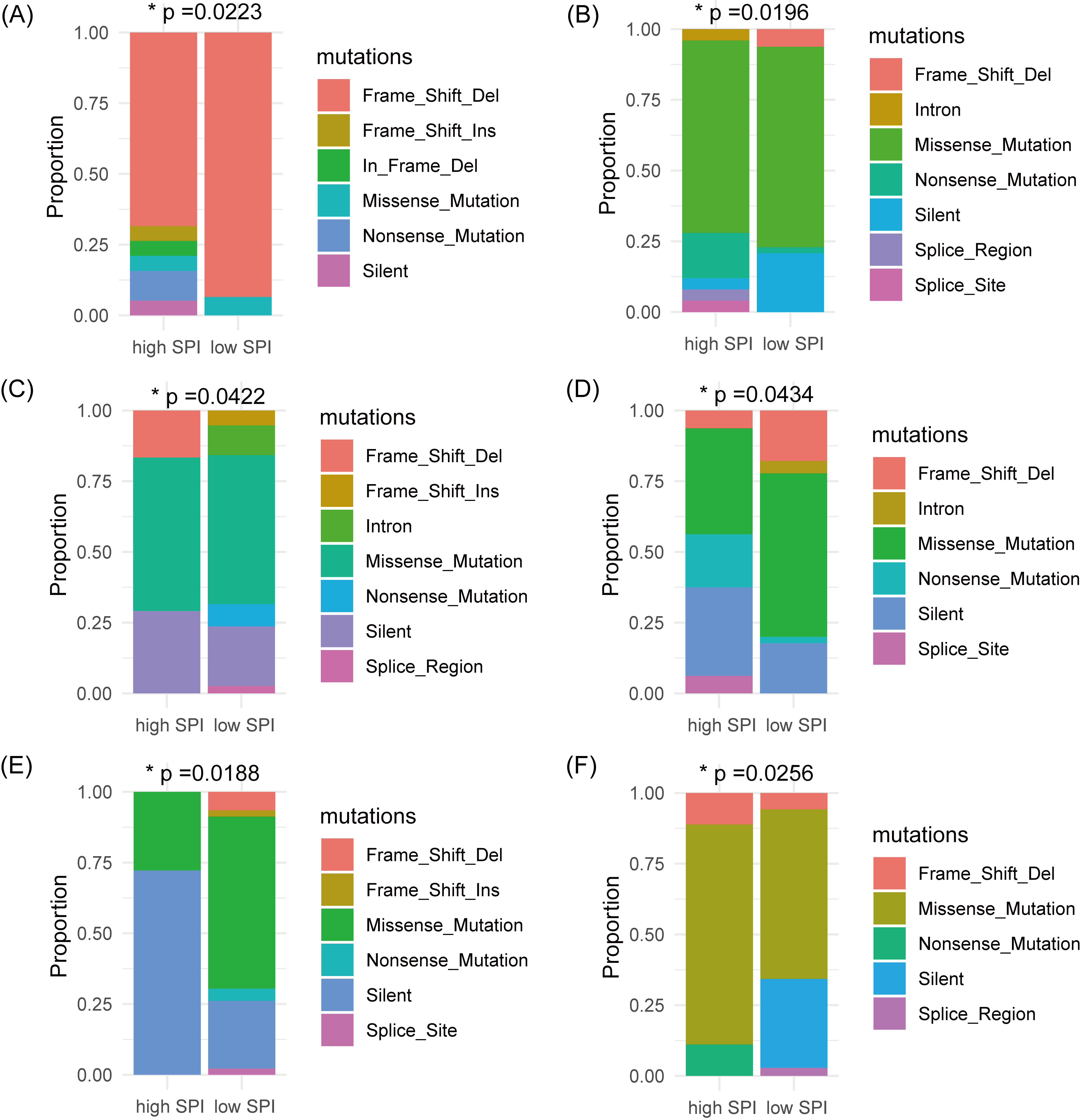


**Supplementary** **Figure S4** Correlation analysis between drug sensitivity, risk score, and associated SPRGs.


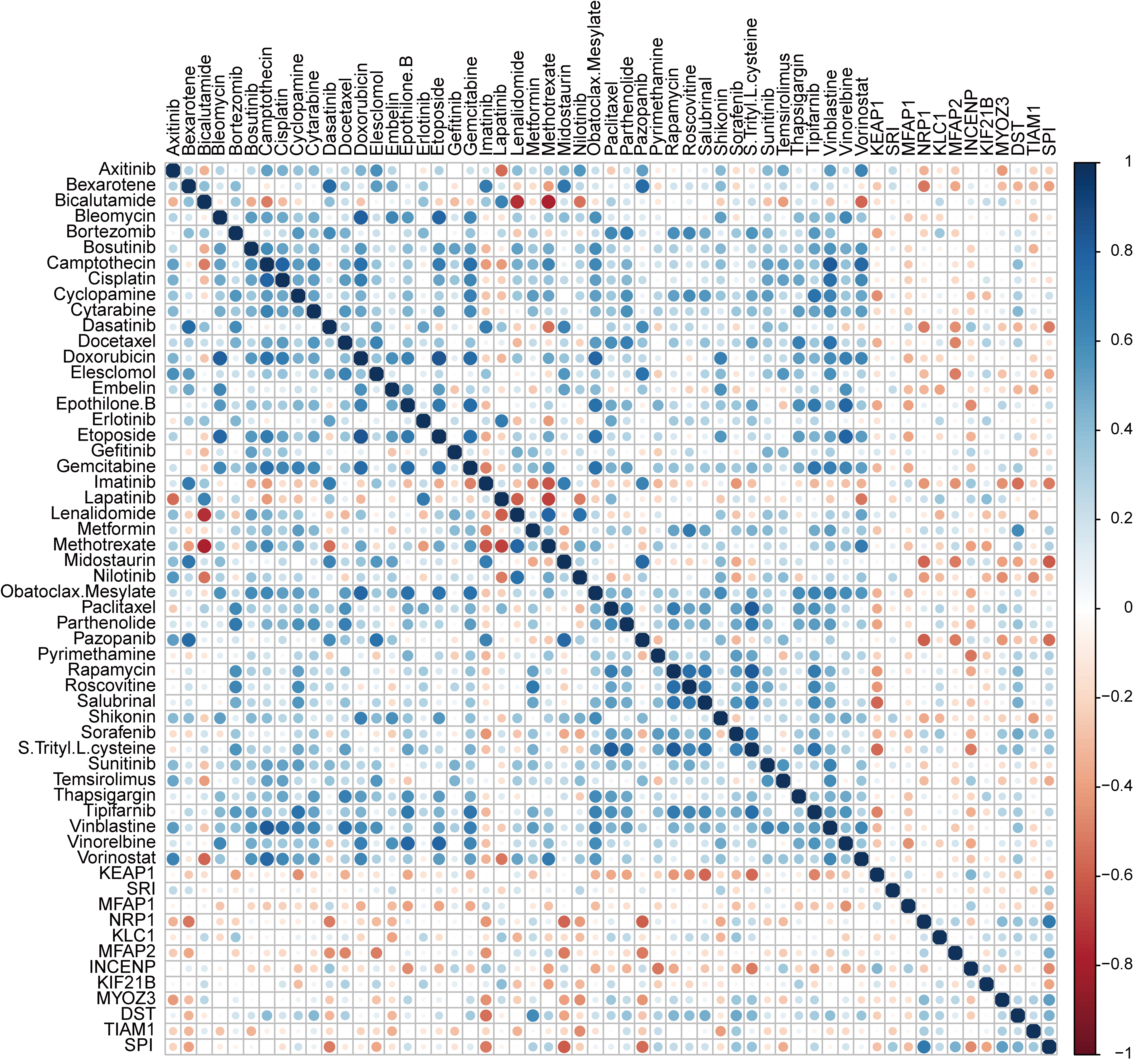

Supplement: Supplementary file 1 [file mmc1.doc]
